# Regulation of *Leishmania* surface coat proteins by the nuclear protein ESB1

**DOI:** 10.1242/jcs.264459

**Published:** 2026-07-13

**Authors:** Jorge Adrián Arias del Angel, Ulrich Dobramysl, Laura Davidson, Richard J. Wheeler, Jack D. Sunter

**Affiliations:** ^1^ School of Biological and Medical Sciences, Oxford Brookes University, Oxford OX3 8DP, UK; ^2^ Peter Medawar Building for Pathogen Research, Nuffield Department of Medicine, University of Oxford, Oxford OX1 3SY, UK; ^3^ Institute of Immunology and Infection, University of Edinburgh, Edinburgh EH9 3FL, UK

**Keywords:** Leishmania, Transcription, Amastin, Cell surface

## Abstract

Unicellular *Leishmania* parasites cause leishmaniases and require a life cycle stage-specific surface coat for pathogenesis. A major protein component of this coat is δ-amastins, which are human infective amastigote life cycle stage-specific transmembrane glycoproteins. *Leishmania* genes are encoded in co-transcribed gene arrays with little gene-specific transcriptional control. Here, we show that the *Leishmania mexicana* ortholog of ESB1 (denoted LmxESB1), a nuclear protein we previously identified in the related parasite *Trypanosoma brucei,* is required for δ-amastin regulation. LmxESB1 localises to a promastigote-specific nuclear body and its deletion caused derepression of δ-amastin expression from specific chromosomal loci. This upregulated δ-amastin phenotype was unstable, recovering over time. Transcriptomic analysis of recovered mutants identified two additional factors – LmxM.34.0190, an NIF-like phosphatase NIFP1, and LmxM.23.0730, the RNA-binding protein RBP10 – where deletion of which also resulted in δ-amastin misexpression. We have therefore identified a novel *Leishmania* nuclear protein that contributes to δ-amastin repression in promastigotes and factors that act in parallel with LmxESB1. This expands our understanding of how *Leishmania* controls stage-specific gene expression of surface coat proteins necessary for pathogenicity.

## Teaser

**Summary:** The surface of the *Leishmania* parasite in the mammalian host is covered by a protein called amastin whose expression is tightly regulated. We show that amastin expression regulation is controlled by the nuclear protein ESB.

## INTRODUCTION

Unicellular *Leishmania* parasites are a family of important human pathogens which cause leishmaniasis, a set of diseases whose symptoms range from a focal cutaneous lesion to life-threatening systemic infection. They are members of the wider group of trypanosomatid parasites, which include the human pathogens *Trypanosoma cruzi* and *Trypanosoma brucei*.

One key adaptation of these parasites conferring pathogenicity is the production of a life cycle stage-specific surface coat. The *Leishmania* cell surface is dominated by lipophosphoglycans, glycoinositolphospholipids, proteophosphoglycans, GP63 and amastin surface proteins (Ferguson, 1997; Jackson, 2010; McConville et al., 2002). Amastins are transmembrane glycoproteins found across the trypanosomatids and distributed across several families (Jackson, 2010). Some of these are encoded as single copy genes or as tandem gene arrays, some are even co-arrayed with the transmembrane protein tuzin. Notably, expansion of the δ-amastin family is associated with evolution of *Leishmania* mammalian parasitism (Jackson, 2010). δ-Amastins are expressed at higher levels in the intracellular amastigote, found within macrophages in the host, than in the promastigote forms in the sand fly vector, and they are necessary for normal virulence (de Paiva et al., 2015).

The well-established view is that regulation of gene expression in trypanosomatids occurs near-exclusively post-transcriptionally (Vanhamme and Pays, 1995). In the genome, protein-coding genes are found in long co-transcribed arrays with a constitutively active promoter (Clayton, 2016). The resulting multi-open reading frame (ORF) nascent transcripts are processed into mRNAs by trans-splicing (Sutton and Boothroyd, 1986), eliminating much possibility of gene-specific transcription level control. This is borne out by numerous studies into trypanosomatid gene expression control including, pertinently, that *Leishmania* GP63 and amastin mRNA are stabilised, contributing to their life cycle stage-specific expression, by specific 3′ UTR sequence elements (Boucher et al., 2002; Brittingham et al., 2001; Rochette et al., 2005), mRNA-binding proteins (Dupé et al., 2014; McNicoll et al., 2005) and mRNA-binding protein-modifying enzymes (Ferreira et al., 2020).

This does not preclude the possibility of targeted control of specific loci in the nucleus. Targeted transcriptional change could hypothetically be achieved by regulated promoters for small transcriptional units, and large transcription units could use alternative internal promoters or early transcription termination. Similarly, a nuclear body could hypothetically degrade or enhance processing of nascent transcript focused on a specific locus, modulating mRNA production.

In *Trypanosoma brucei*, both transcriptional control and focused RNA degradation are associated with surface coat protein expression. It has strong stage-specific regulation of the RNA polymerase I (Pol I) promoters for the transcription units, which include the procyclin or variant surface glycoprotein (VSG) surface coat protein genes (Günzl et al., 2003). Procyclin, for the procyclic (insect) life cycle stage, is found in a normal diploid chromosomal locus, containing procyclins and procyclin-associated genes (PAGs) (Haenni et al., 2006). VSG, for metacyclic and bloodstream form stages, is found in haploid sub-telomeric loci, containing one VSG and, for bloodstream form expression sites, VSG expression site-associated genes (ESAGs) (Hertz-Fowler et al., 2008). We recently discovered the first transcriptional activator of VSG expression sites, ESB1 (López-Escobar et al., 2022), which localises to the nuclear expression site body (ESB) (Navarro and Gull, 2001). The ESB also uses focal control of RNA degradation to modulate the output of these transcription units. VSG mRNA (and consequently, VSG protein) are much more abundant than ESAGs mRNAs. This is achieved by a combination of very long VSG mRNA half-life conferred by mRNA-binding proteins (Melo do Nascimento et al., 2021), and the degradation of some ESAG transcript at the site of transcription by ESB2 (Lansink et al., 2026).

Here, we sought to test whether previously unrecognised nuclear bodies contribute to GP63 or amastin expression control in *Leishmania.* There is no evidence for trypanosomatids outside of the African trypanosomes, including *Leishmania*, having similar specialised surface coat protein transcription units. Nor is there evidence for use of Pol I promoters for protein-coding gene expression. However, all trypanosomatid parasites have an ESB1 ortholog, which is necessary for the *T. brucei* ESB to form (López-Escobar et al., 2022), potentially indicating evolution from an ancestral ability to form nuclear bodies and focused regulation of transcription and RNA degradation.

Here, we show that *Leishmania* ESB1 is a promastigote life-cycle stage-specific nuclear factor necessary for normal repression of specific δ-amastin loci. Defective amastin expression in ESB1 deletion mutants was unstable and recovered over time, and we identified the RNA-binding protein LmxM.23.0730 (RPB10) and LmxM.34.0190 (NIF-like phosphatase, phosphatase domain only) as amastin-regulating factors whose altered expression contributed to the amastin expression defect recovery. We have therefore shown that an ESB1-containing nuclear structure contributes to regulation of stage-specific δ-amastin expression in *Leishmania* and identify proteins whose altered expression can compensate for this defect.

## RESULTS

**Fig. 1.**
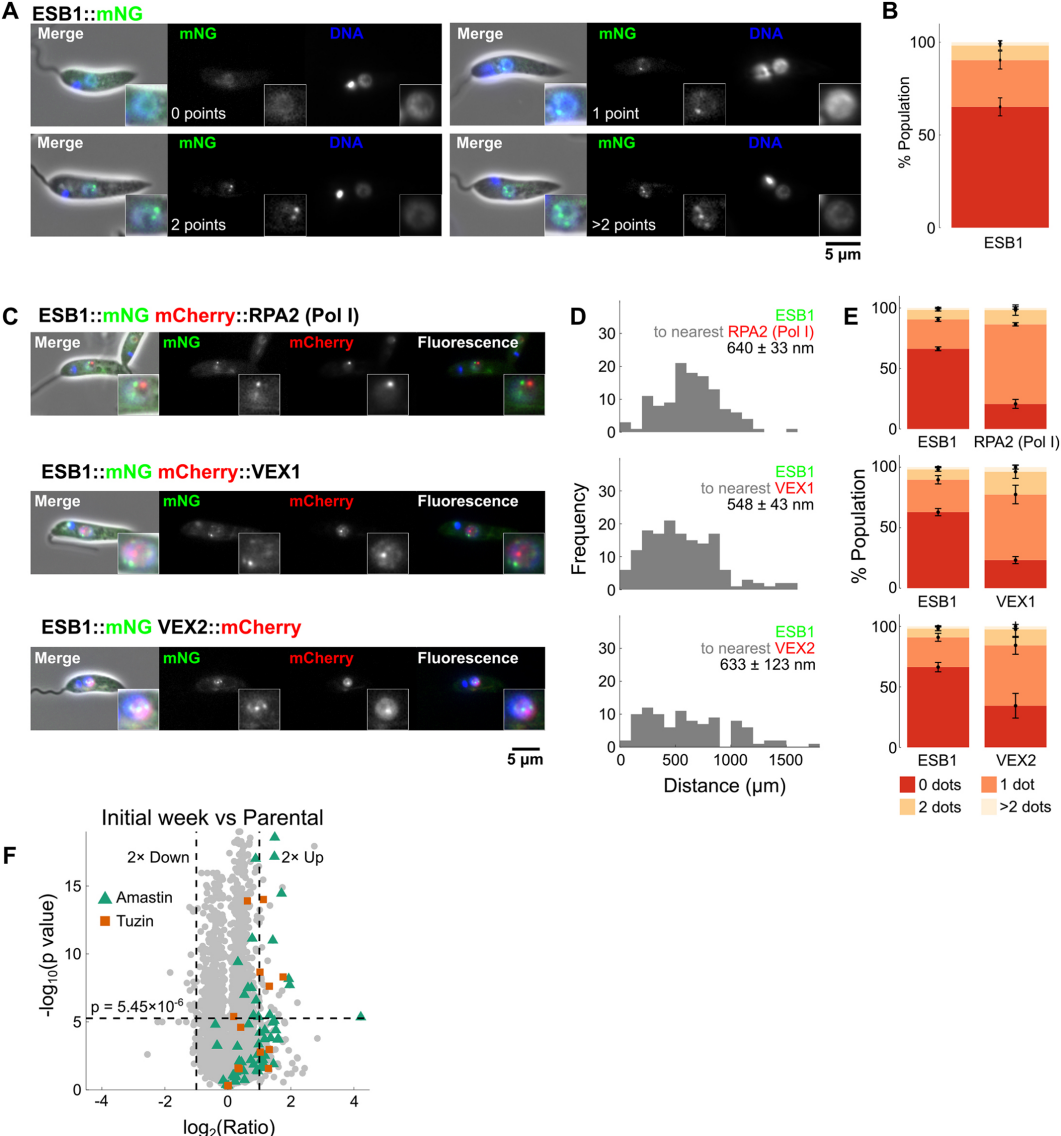
***Leishmania* ESB1 is a nuclear protein expressed in the promastigote necessary for normal promastigote amastin expression.** (A) Subcellular localisation of LmxESB1 in promastigotes. Representative fluorescence micrographs of promastigotes expressing ESB1::mNG showing cells with 0, 1, 2 or more bright fluorescence foci. Separate mNG and DNA fluorescence images are shown, along with an overlay with phase contrast. A detailed view of the nucleus is in the inset. (B) The proportion of cells with different numbers of bright nuclear foci shown on the right (680 cells from three technical replicates were analysed from exponentially growing population). (C) Position of ESB1 foci relative to RNA polymerase I (RPA2 subunit), VEX1 and VEX2 foci. Representative fluorescence micrographs of promastigotes expressing ESB1::mNG and either mCh::RPA2, mCh::VEX1 or VEX2::mCh showing cells with one or more foci of both tagged proteins. Separate mNG and mCherry fluorescence images are shown, along with an overlay of all fluorescence images, including DNA stain, and an overlay with phase contrast. A detailed view of the nucleus is in the inset. (D) Histogram of the shortest distance between signal foci of ESB1::mNG and the mCherry-tagged second protein, measured from cells with at least one focus visible for both tagged proteins (ESB1::mNG-mCherry::Pol1, *n*=120; ESB1::mNG-mCherry::VEX1, *n*=154; ESB1::mNG-VEX2::mCherry, *n*=95). (E) The proportion of cells with different numbers of bright nuclear foci (ESB1::mNG-mCherry::Pol1, *n*=410; ESB1::mNG-mCherry::VEX1, *n*=412; ESB1::mNG-VEX2::mCherry, *n*=632; cells were analysed across three technical replicates from exponentially growing populations). (F) Plot of the ratio of transcript abundance in the parental cell line to the ESB1 deletion mutants against statistical significance of abundance change. Each data point represents a single transcript, average of n=3 clonal deletion populations. The horizontal dashed line represents *p=*0.05 with Bonferroni multiple comparison correction (*p*=5.45×10^–6^, considering 9182 total hypotheses), the vertical dashed lines represent two-fold increased or decreased transcript abundance. Amastin and Tuzin genes are highlighted. For clarity plotting highly upregulated transcripts around the significance threshold the y axis is truncated. For extended plots focusing on statistically significant smaller fold changes see Fig. S1I. Error bars in B and E show mean and 95% c.i.

Given the conservation of full-length orthologs of *T. brucei* ESB1 (TbESB1) across most trypanosomatids (López-Escobar et al., 2022), we asked whether *L. mexicana* ESB1 (LmxESB1) had a similar localisation to TbESB1. We generated promastigote cell lines expressing LmxESB1 endogenously tagged at the C-terminus with mNeonGreen (mNG) and examined the localisation of the fusion protein by fluorescence light microscopy. Like TbESB1, LmxESB1::mNG had a nuclear localisation (Fig. 1A) – it localised to a subnuclear focus visible in ∼40% of G1 cells, most often a single focus (Fig. 1B). A similar pattern was seen during the rest of the cell cycle (Fig. S1A). Differentiating the LmxESB1::mNG-expressing cell line into axenic amastigotes revealed a similar localisation in amastigotes when detectable (Fig. S1B), but far fewer cells (<5%) had any detectable signal (Fig. S1C). LmxESB1 therefore appears to form a promastigote-specific nuclear body.

In *T. brucei* bloodstream forms, TbESB1 is positioned close to a set of proteins involved in normal VSG expression regulation, RNA Pol I, VEX1 and VEX2 (Faria et al., 2019; Glover et al., 2013; López-Escobar et al., 2022). To determine whether LmxESB1 is associated with these proteins in *Leishmania*, we analysed the localisation of LmxESB1 relative to RPA2 (an RNA Pol I subunit) and the *L. mexicana* orthologs of VEX1 and VEX2. mCh::RPA2 localised predominantly to a single focus adjacent to the nucleolus, whereas mCh::VEX1 and VEX2::mCh localised to the nucleoplasm with most cells also containing at least one bright nuclear focus (Fig. 1C,E). We did not observe any close association between LmxESB1 foci and those of RPA2, VEX1 or VEX2, with the nearest focus being on average ∼550 nm distant (Fig. 1D). Unlike TbESB1, this shows that LmxESB1 localises to distinct nuclear sub-domains from these proteins. As LmxESB1 did not colocalise with RNA Pol I, we investigated whether LmxESB1 colocalised with RNA Pol II. We endogenously tagged RPB2 (RNA Pol II subunit) and analysed its localisation relative to LmxESB1 (Fig. S1D). Again, we did not observe any close association between LmxESB1 foci and those of RPB2. For these localisation analyses, the correct integration of the genes encoding the mNG and mCherry (mCh) fluorescent proteins was confirmed by PCR (Fig. S1E–H).

Given its role of TbESB1 protein in transcriptional activation, to gain insight into the function of LmxESB1 we deleted both alleles using CRISPR/Cas9-mediated genome modification and analysed the transcriptomic changes in three clonal populations. RNA samples were taken as soon as they had grown to a population of ∼10^8^ cells (10 ml of late-exponential phase culture) after transfection. Our transcriptomic analysis confirmed the deletion of the LmxESB1 coding sequence (Fig. S2A). The cells were morphologically promastigote, with an elongated cell body and long motile flagellum, but LmxESB1 deletion caused a specific set of multiple comparison-corrected significant changes in transcript level (*P*=0.05/9182=5.45×10^–6^); 45 transcripts were upregulated and 16 downregulated (Fig. 1F; Table S1). Of the 16 genes downregulated there are few commonalities except for five genes associated with protein translation (LmxM.27.1710, LmxM.36.3880, LmxM.36.6980, LmxM.29.3240 and LmxM.34.1430), which likely reflects the slow growth of these cells. Notably, the upregulated set included many δ-amastin (*n*=8) and tuzin (*n*=4) transcripts (Fig. S2B; Table S1), which are the dominant surface coat protein of the mammalian-infective amastigote stage. Significantly upregulated non-amastin non-tuzin transcripts also tended to be amastigote-associated, such as cathepsin L protease (*n*=3), hydrophilic acylated surface protein a (HASPa; *n*=1) and ascorbate peroxidase (*n*=1). This indicates that LmxESB1 is a negative regulator of specific amastigote-associated transcripts, most prominently a subset of δ-amastins (Fig. S2B; Table S1).

To confirm that this transcriptome phenotype was not an aberrant effect of gene deletion, perhaps associated with cell stress from drug selection and perturbations in cell function, we deleted FAZ5 and analysed its transcriptome using the same pipeline (Fig. S3A–D; Table S2). FAZ5 is a well-studied cytoskeletal protein that is important for cell morphogenesis but does not have a role in gene regulation. This caused few significant changes in transcript abundance (Fig. S3B), which did not strongly correlate with the LmxESB1 deletion (Fig. S3C) and did not include any amastins or tuzins (Fig. S3C,D). This demonstrates that changes in amastin expression were specific to LmxESB1 deletion and not a generic stress response.

**Fig. 2.**
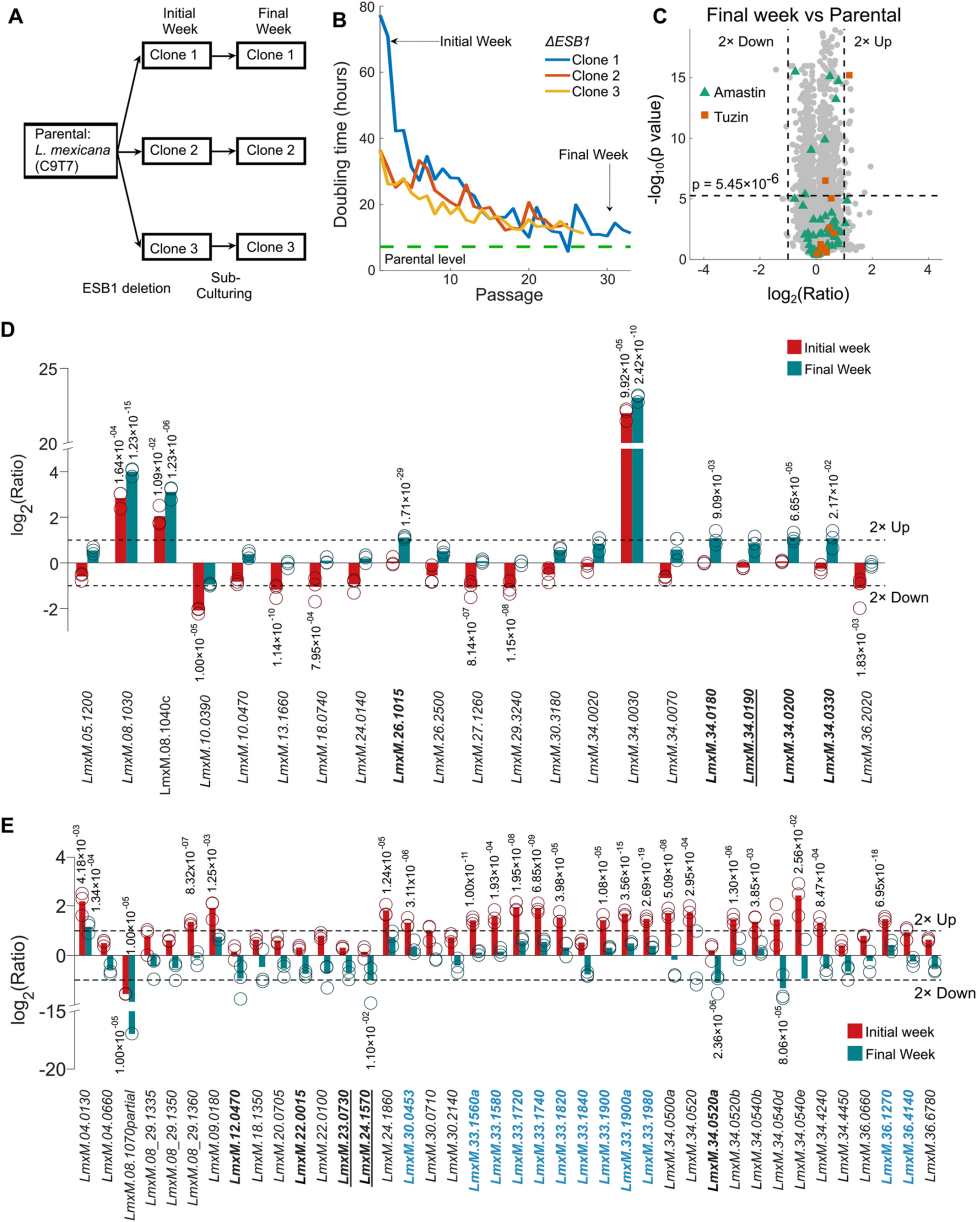
**The aberrant amastin expression phenotype due to LmxESB1 deletion recovers over time with associated transcript abundance changes.** (A) Diagrammatic representation of the strategy for LmxESB1 deletion mutant generation and maintenance in culture; DNA and RNA samples were taken in the initial (once recovered from transfection and drug selection) and final week (once growth rate had reached near parental). (B) Graph of population doubling time, a measure of growth rate, over time for the three clonal LmxESB1 deletion mutants. Each data point represents a single measurement of population growth rate from a 24 h interval for each clonal deletion population. The horizontal dashed line represents the parental cell line doubling time (7.2 h), the mean of *n*=3 measurements. Measurements stopped as soon as the doubling time reached a stable value. (C) Plot of the ratio of transcript abundance in the parental cell line to the LmxESB1 deletion mutants, sampled in the final week once growth rate had recovered, against statistical significance of abundance change. Each data point represents a single transcript, average of *n*=3 clonal deletion populations The horizontal dashed line represents *P=*0.05 with Bonferroni multiple comparison correction (*P*=5.45×10^−6^, considering 9182 total hypotheses), the vertical dashed lines represent two-fold increased or decreased transcript abundance. Amastin and Tuzin genes are highlighted. (D) Changes in all transcripts that showed statistically significant (P<0.05, two tailed paired *t*-test) upregulation in the final week after growth rate recovery in comparison to the initial week post-transfection, plotting the ratio compared to the parental cell line. Data points represent each of the three LmxESB1 deletion clonal populations, bars represent the mean. *P*-values indicate statistically significant changes from the parental cell line. (E) As for D, but for all transcripts that showed statistically significant downregulation in the final week after growth rate recovery in comparison to the initial week post transfection. In D and E, genes that changed their expression pattern (from basal to upregulated or from basal to downregulated) are highlighted in bold. Among these, three are RNA regulators (highlighted in bold and underlined) and were selected for further analysis. Amastin genes are highlighted in blue; however, no tuzin genes were significantly modified during this period.

After generating the LmxESB1 clonal deletion populations, we grew them by repeated subculture in promastigote culture conditions under continuous drug selection (Fig. 2A); although they initially had a severe growth rate defect, we saw a dramatic increase in growth rate over time. After 7 weeks, the growth rate of all three clonal deletion populations was similar to that of the parental cell line (Fig. 2B). To determine whether the transcriptome phenotype had also returned to normal, we compared the transcriptome of the LmxESB1 deletion mutants after growth recovery with the parental levels (Fig. 2C; Table S3). This showed that only one tuzin gene remained upregulated compared to the parental cells, indicating a return to the parental phenotype.

Given the recovery to the parental phenotype, we wanted to examine whether there were any genomic changes which were compensating for the loss of LmxESB1. Mutations to coding sequences might identify proteins regulating amastin expression. We mapped single nucleotide polymorphisms altering protein coding sequence and insertions or deletions in protein coding sequence. Over the period as the growth of the mutants recovered the genome sequence was remarkably stable, and we did not identify any genes that were hotspots for changes in the protein coding capacity because of LmxESB1 deletion (Fig. S3E) and we saw similar stability for the LmxFAZ5 deletion clones (Fig. S3F). There was one mutation missense-variant (c.238A>G) shared by all the LmxESB1 deletion mutants, which was in the gene (LmxM.10.0470), encoding one of the copies of GP63. Given this mutation was in one copy of gene present in an array that encodes a surface protein, we do not think it is likely to have a role in regulating amastin expression.

As genomic changes could not explain the recovery of the LmxESB1 deletion mutant phenotype, we carried out a detailed comparison of the transcriptomes of the LmxESB1 deletion clonal populations from the initial week post transfection with the corresponding clonal transcriptome after growth recovery (Table S3). We identified those genes which, after recovery, had either significantly increased (*n*=21) (Fig. 2D) or decreased (n*=*40) transcript abundance (Fig. 2E), and as expected, the decreased set includes 12 amastin transcripts (Fig. S2B; Table S3). Among the other transcripts, several had a return to an abundance closer to the parental cells (Fig. 2D); for example, LmxM.36.2020 (chaperonin HSP60, mitochondrial precursor), LmxM.18.0740 (elongation factor Tu, mitochondrial), LmxM.29.3240 (glutamyl-tRNA synthetase), LmxM.13.1660 (chaperonin TCP20), and LmxM.05.1200 (a prefoldin subunit), were initially downregulated in comparison to the parental cells. These transcripts are associated with cell and mitochondrial protein production and their initial drop followed by an increase likely reflects the initial slower growth of the LmxESB1 deletion mutants, which then recovers.

The best candidates involved in recovering the LmxESB1 deletion phenotype are the upregulated genes and downregulated genes encoded by transcripts that had only minimally changed between the parental cells and in the initial week post-transfection, as this indicated a change in expression during the recovery period (Fig. 2D,E). Our analysis method considered the average of all three independent clones, biasing candidate identification to changes that were common to all three. It is notable that all three independent clones had consistent changes across several transcripts. We also noted that three such transcripts were positioned directly either side of a diverging strand switch region (Fig. S4A). In trypanosomatid parasites, including *Leishmania,* these are the areas of RNA Pol II transcription initiation – there were no genome sequence changes in this region, suggesting an epigenetic change. We examined the encoded domains and annotations of the up- and down-regulated genes to identify those with a biologically plausible role in gene regulation, selecting three candidates for further analysis: LmxM.34.0190, LmxM.24.1570 and LmxM.23.0730. LmxM.24.1570 and LmxM.23.0730 are orthologs of *T. brucei* DRBD13 and RBP10, respectively, and were downregulated during recovery. Both are predicted to encode RNA-binding domains, indicative of a potential role in transcript regulation, and are both involved in stage-specific mRNA stability in *T. brucei* (Jha et al., 2015; Mugo and Clayton, 2017). LmxM.34.0190 was upregulated during recovery and it is an NLI-interacting factor-like phosphatase (which we call NIFP1). This is a large family of phosphatases, and is intriguing given that NLI-interacting factor is involved in the regulation of RNA Pol II and transcription factors (Hausmann and Shuman, 2003); however, the phosphatase domain is just one functional domain of this protein.

**Fig. 3.**
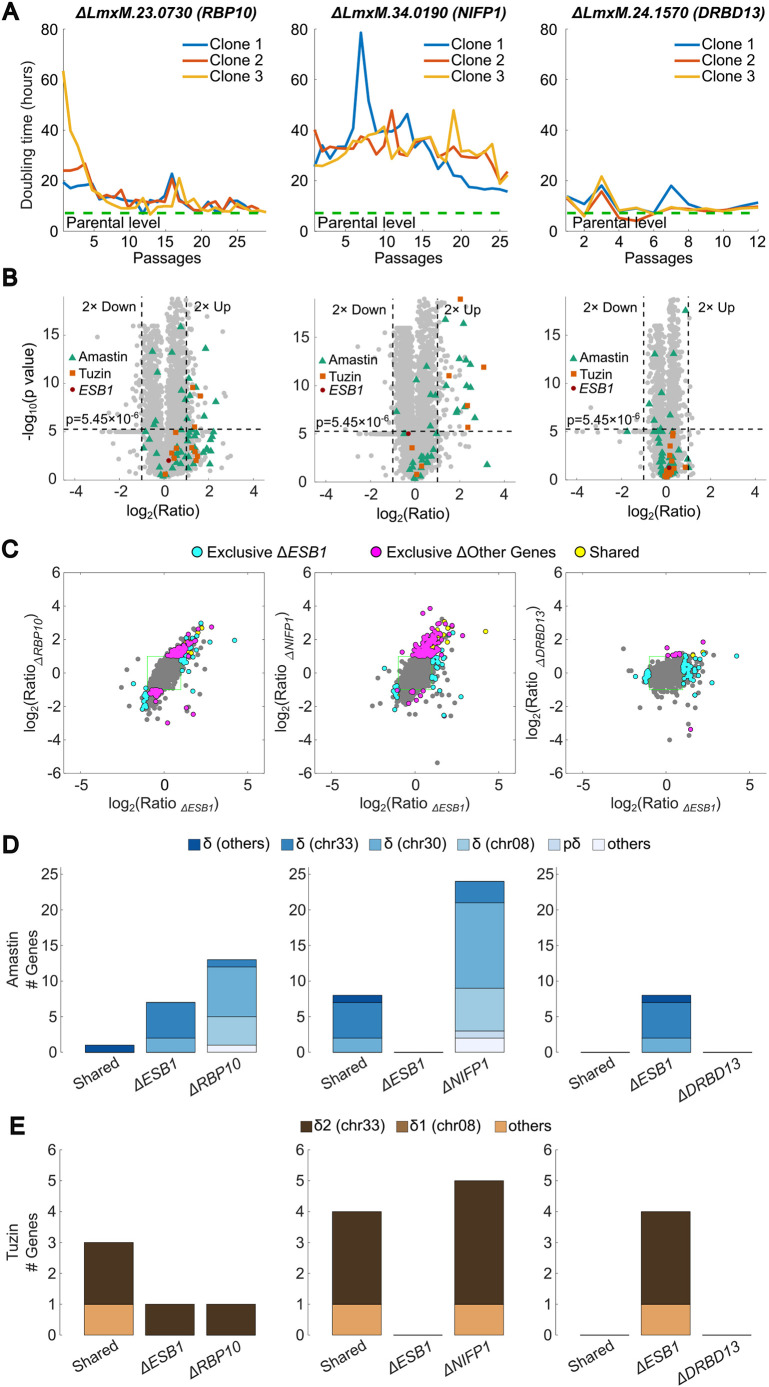
**LmxRBP10 and LmxNIFP1 encode proteins necessary for normal δ-amastin expression.** (A) Graph of population doubling time, a measure of growth rate, over time for the LmxRBP10, LmxNIFP1 and LmxDRDB13 deletion mutants. Each data point represents a single measurement of population growth rate from a 24 h interval for each mutant. The horizontal dashed line represents the parental cell line doubling time (7.2 h), the mean of *n*=3 measurements. (B) Plot of the ratio of transcript abundance in the parental cell line to the LmxRBP10, LmxNIFP1 and LmxDRBD13 deletion mutants, against statistical significance of abundance change. Each data point represents a single transcript, average of *n*=3 clonal deletion populations The horizontal dashed line represents *P=*0.05 with Bonferroni multiple comparison correction (*P*=5.45×10^–6^, considering 9182 total hypotheses), the vertical dashed lines represent two-fold increased or decreased transcript abundance. Amastin and tuzin genes are highlighted. For clarity, plotting highly upregulated transcripts around the significance threshold the *y*-axis is truncated. For extended plots focusing on statistically significant smaller fold changes see Fig. S4E. (C) Correlation of the fold change of transcript abundance following LmxRBP10, LmxNIFP1 or LmxDRBD13 deletion in comparison to the parental cell line, plotted against fold change of transcript abundance following ESB1 deletion compared to the parental cell line. Each data point represents the mean from *n*=3 clonal deletion mutant populations. Transcripts statistically significantly up- or down-regulated by at least twofold are highlighted, colour coded by whether they have altered expression in one or both deletion mutant clonal populations. The green box represents transcripts with less than two-fold increase or decrease. Cyan, magenta and yellow data points represent transcripts significantly changed in only ESB1 deletion relative to parental, the other deleted gene relative to parental or both, respectively. (D) Number of significantly upregulated amastin transcripts from B, categorised by change in one or both mutant cell lines, broken down by type – for δ-amastins, and which chromosome (chr) they are encoded on, proto-δ-amastins (pδ) and other (amastin families α, β or γ). (E) Number of significantly upregulated tuzin transcripts from B, broken down by which chromosome they are encoded on.

To determine whether these three candidates had a role in amastin gene regulation, we deleted each gene individually and analysed the growth (Fig. 3A) and transcriptome of the deletion mutants immediately after recovery from transfection (Fig. 3B, Tables S4–S6). Deletion of candidate genes was confirmed by loss of RNA sequencing reads aligning to the coding sequences for these genes (Fig. S4B).

Both the NIFP1 and RBP10 deletion mutants were slow growing, whereas the DRBD13 deletion mutant was not (Fig. 3A). Analysis of the deletion mutant transcriptomes showed that the slow growth of RBP10 and NIFP1 deletion mutants both correlated with an increase of amastin and tuzin transcripts, whereas the LmxDRBD13 deletion mutant had no large transcriptome changes (Fig. 3B; Tables S4–S6). In none of the mutants was there a significant change in LmxESB1 transcript abundance (Fig. 3B; Tables S4–S6). RBP10 and NIFP1 are therefore also acting as negative regulators of amastin expression in the promastigote, acting in parallel to LmxESB1.

To compare the transcriptomic changes of each deletion mutant to the changes observed in the LmxESB1 deletion mutant, we plotted the fold change for each transcript relative to the parental cells for each deletion mutant (RBP10, NIFP1 and DRBD13) against the fold change for the same transcript in the LmxESB1 deletion mutant (initial week after transfection) (Fig. 3C). For the RBP10 and NIFP1 deletion mutants there was a good correlation with the LmxESB1 deletion mutants, with transcripts upregulated in the LmxESB1 mutant also upregulated in these mutants. Conversely, there was a poor correlation between the DRBD13 deletion mutant and the LmxESB1 deletion mutant, which was expected due to the limited transcriptomic changes in this mutant (Fig. 3C).

There were, however, clear differences between the mutants. We determined which transcripts were statistically significantly upregulated at least twofold and were upregulated in both the NIFP1 and LmxESB1 or both the LmxRBP10 and LmxESB1 deletion mutants (Fig. 3C, Tables S4–S6). As these common upregulated transcripts were mainly amastins and tuzins, we plotted just those gene families (Fig. 3D,E). This showed a clear pattern of which δ-amastins were upregulated in the different deletion mutants. δ-amastins are a multi-copy gene family found predominately within three gene arrays on chromosome 8, 30 and 33, with additional copies around the genome (Fig. S2B). Deletion of LmxESB1 and LmxRBP10 led to upregulation of distinct sets of amastin transcripts. Those in the LmxESB1 deletion mutant mostly derived from a single δ-amastin array on chromosome 33, with additional ones from chromosome 8. Those in the LmxRPB10 deletion mutant mostly derived from chromosome 30 and 8 (Fig. 3D). In comparison, amastin transcripts from across the genome were upregulated in the NIFP1 deletion mutant, including all those upregulated in the LmxESB1 deletion mutant (Fig. 3D). Most tuzin genes are found co-arrayed with δ-amastins in one locus on chromosome 33 and another on 8, with additional copies elsewhere in the genome. The tuzins showed a different pattern with upregulated tuzin transcripts generally shared between the deletion mutants, particularly for RPB10 and LmxESB1 (Fig. 3E). Tuzin transcripts originating from chromosome 8 were not upregulated in any of these mutants. Similar comparison of the RBP10 and NFIP1 deletion mutants (Fig. S4C,D) completes this picture. This suggests a layer of regulation post-transcriptionally by at least one of the two deleted genes under comparison, decoupling amastin and tuzin transcript abundance from the co-arrayed chromosome 33 and 8 loci.

To determine whether RBP10, NIFP1 and DRBD13 also functioned in the nucleus like LmxESB1, we tagged them on the C-terminus with mNG (Fig. S5A–C), with successful integration of the mNG tag confirmed by PCR (Fig. S5D). Both DRBD13 and NIFP1 were found in the cytoplasm, with NIFP1 showing an enrichment around the basal body (Fig. S5B,C). The signal from the cells in which RBP10 had been endogenously tagged was weak with a reticulated pattern, typical of background autofluorescence and indicative of low expression (Fig. S5A). Taken together, this suggests that these proteins function outside of the nucleus.

## DISCUSSION

We showed that, surprisingly, the *Leishmania* ortholog of ESB1 is a nuclear protein necessary for normal expression levels of *Leishmania* surface coat proteins. This was unexpected, as while there is extensive evidence for regulation of transcript abundance post-transcriptionally, plausibly sufficient for stage-specific amastin expression (Boucher et al., 2002; Brittingham et al., 2001; Dupé et al., 2014; Ferreira et al., 2020; McNicoll et al., 2005; Rochette et al., 2005), there is no evidence for *T. brucei-*like RNA Pol I expression of surface coat protein genes in *Leishmania*. Nonetheless, LmxESB1 localises to nuclear focus and is specifically necessary for repression of expression of δ-amastin gene predominantly on chromosome 33. Although the amastin and tuzin genes form the largest cluster of those transcripts significantly changed after LmxESB1 deletion, there were additional transcripts upregulated that are associated with amastigotes, including three cathepsin genes and the ascorbate peroxidase gene (Pal et al., 2010; Somanna et al., 2002). This suggests that LmxESB1 might have a broader role in amastigote gene regulation beyond amastins and tuzins.

When we consider the potential mechanism of the nuclear-localised LmxESB1, local chromosome organisation suggests that LmxESB1 does not act as a transcriptional activator. The δ-amastins on chromosome 33 whose expression levels were changed by LmxESB1 deletion are found towards the centre of a region of similarly oriented genes. This is likely to be a single transcription unit, and we did not identify significant change in amastin-flanking gene transcripts in the absence of LmxESB1, although there could be an internal promoter. This suggests that LmxESB1 instead contributes to immediate nascent transcript degradation or to repression of effective δ-amastin transcript processing from this locus, although LmxESB1 lacks a nuclease domain or similar that could do so directly. Whatever the mechanism, the identification of LmxESB1-containing nuclear bodies as having a role in surface coat protein expression control in *Leishmania* was unexpected and suggests that they have a similar role across trypanosomatids. Future work will be required to determine the specific mechanism of LmxESB1 and whether it engages directly with the DNA on chromosome 33 and elsewhere in the genome, and its interacting partners.

By searching for transcripts that change abundance associated with recovery of the LmxESB1 deletion mutant phenotype over time, we identified NIFP1 and RBP10 as having amastin expression regulation functions. NIFP1 is clearly a negative regulator of amastin expression: first, because an increased expression of NIFP1 was associated with recovery of the promastigote LmxESB1 deletion phenotype and, second, because its deletion led to increased abundance of many δ-amastins. RBP10 deletion showed that it is also a negative regulator of amastin expression. However, its contributions are likely more complex, as RBP10 transcript decreased in abundance during recovery of the promastigote LmxESB1 deletion phenotype – when increased expression would be predicted to help overcome loss of repression of amastin expression. DRBD13 transcript similarly decreased in abundance but was not directly necessary for normal amastin transcript abundance regulation. We suggest that NIFP1 is an upstream master regulator of δ-amastin transcript abundance, and that transcript abundance of RNA-binding proteins implicated in life cycle stage-specific stability [based on their function in *T. brucei* (Jha et al., 2015; Mugo and Clayton, 2017)] were able to become dysregulated as NIFP1 transcript abundance increased following LmxESB1 deletion.

NIFP1 is a small phosphatase, and a member of the family of phosphatases that regulate RNA Pol II activity (including FCP1 in yeast and CTDP1 in humans) although as a single domain of a larger protein (Hausmann and Shuman, 2003). The severe growth rate defect following its deletion points to a wider function than just surface transcript abundance control, but nonetheless is informative for the wider network of factors contributing to control of amastin transcript abundance.

When tagged with mNG, NIFP1 and RBP10 did not localise to the nucleus; therefore, it is unlikely that these proteins directly interact with LmxESB1. This suggests that these proteins operate in the cytoplasm, regulating transcript abundance. Moreover, our identification of proteins that do not directly interact with LmxESB1 but operate within the same transcript regulation pathway, shows the power of careful interrogation of transcript changes during deletion mutant recovery. This will open additional experimental avenues to unpick complex gene regulatory pathways, complementing approaches that focus on direct protein interactions.

Through this work, we have discovered that a nuclear body containing LmxESB1 is necessary for normal repression of δ-amasin expression in *Leishmania* promastigotes. Adaptation of the LmxESB1 deletion mutant over time in culture allowed us to identify additional factors controlling amastin transcript abundance whose transcript abundance changes compensated for loss of LmxESB1, revealing new contributions to this regulatory network.

## MATERIALS AND METHODS

### Parasite strain and cell culture

*L. mexicana* promastigotes constitutively expressing T7 RNA polymerase and Cas9 and Hyg and NAT selectable markers (C9T7) (Beneke and Gluenz, 2019) were cultured in M199 medium (Invitrogen, cat. no. 31100-019) supplemented with 10% heat-inactivated fetal calf serum (FCS; Invitrogen, cat. no. 10500064) at 28°C. Cell lines were monitored for contamination, including mycoplasma contamination, through DNA staining and microscopy. Prior to starting experimental work, the C9T7 strain was cloned by limiting dilution (see below) and subject to genome and transcriptome sequencing to give a single clonal parental population with a reference genome and transcriptome for all subsequent analysis (Zenodo doi:10.5281/zenodo.20920273). Promastigote cultures were maintained by subculture upon reaching a cell density of ∼10^7^ cells/ml and were used up to passage 15. Axenic amastigotes were generated by incubating 0.5×10^7^–1.0×10^7^ promastigotes in Schneider's medium (pH 5.5) at 32°C for 72 h (Bates, 1994). Cell density was determined using a Beckman Coulter Counter Z1.

### Endogenous tagging

Constructs were designed with CRISPR-assisted modification of one allele of genes to achieve endogenous tagging with a fluorescent protein, using a PCR-based strategy for construct generation. All primers were designed according to Beneke and Gluenz (2019) (sequences in Table S7), and constructs generated using PCR as described in Beneke and Gluenz (2019), using the pLPOT plasmid series (Edwards et al., 2018). The localization of LmxESB1 (LmxM.34.0660) was determined by fusing one allele of the protein coding gene at its C terminus with the fluorescent protein mNeonGreen (mNG) using BSR/Blasticidin S hydrochloride for selection. Colocalisation was determined by tagging mCherry (mCh) fluorescent protein using PAC/Puromycin dihydrochloride for selection. For VEX1 (LmxM.31.3090) and RPA2 (LmxM.25.0620, Pol I subunit), the fluorescent protein tagging was performed at the N terminus, whereas for VEX2 (LmxM.09.1240) and RBP2 (LmxM.30.0160), it was done at the C terminus. For LmxRBP10 (LmxM.23.0730), LmxNIFP1 (LmxM.34.0190) and LmxDRBD13 (LmxM.24.1570), the fluorescent protein tagging was performed at the C terminus. gDNA was isolated from cell lines using the Qiagen DNeasy Blood and Tissue kit.

### Gene deletion

Constructs were designed for CRISPR-assisted replacement of both alleles of a gene with a drug selectable marker, using a PCR-based strategy for construct generation. All primers were designed according to Beneke and Gluenz (2019) (sequences in Table S7), and constructs generated using PCR and the pT plasmid series (Beneke and Gluenz, 2019). LmxESB1 (LmxM.34.0660), LmxFAZ5 (LmxM.36.5970), LmxRBP10 (LmxM.23.0730), LmxNIFP1 (LmxM.34.0190) and LmxDRBD13 (LmxM.24.1570) were deleted using BSR/blasticidin S hydrochloride and PAC/puromycin dihydrochloride for selection.

### Electroporation and drug selection

Prior to electroporation, cells were maintained under 32 μg/ml hygromycin B (hygromycin resistance gene, Hyg) and 50 μg/ml nourseothricin sulfate (nourseothricin N-acetyltransferase, NAT) to ensure expression of Cas9 and T7 polymerase. PCR-generated constructs and sgDNAs (1–5 μg) were purified by ethanol precipitation and mixed with 10^7^ cells in 100 μl of Tb-BSF buffer (Schumann Burkard et al., 2011) for transfection. Electroporation was performed using the Amaxa Nucleofector IIb system (program X-001, Lonza) with 2-mm-gap cuvettes (Dean et al., 2015). Following transfection, cells were transferred to 10 ml of prewarmed medium and incubated for 6 h before the addition of selection drugs using the appropriate combination of 10 μg/ml blasticidin S hydrochloride (blasticidin S resistance gene, BSR; Melford Laboratories), 20 μg/ml puromycin dihydrochloride (puromycin-N-acetyltransferase gene, PAC; Melford Laboratories).

For protein localisation analysis, non-clonal populations were analysed. For deletion mutant analysis, clonal cell lines were established by limiting dilution in 96-well plates in the presence of selection drugs as described in Dean et al. (2015), and three clones were recovered and expanded for genomic and RNA extraction for sequencing and for subsequent experiments.

The selected clones were maintained through continuous passaging for the knockout experiments, and the characteristic doubling time of each clone was determined. For this, the cell density was measured both before (C_f_, final density) and after (C_i_, initial density) each passage, along with the corresponding incubation periods (*t*). The doubling time was then calculated by *t*×log_10_(2) / log_10_(C_f_ / C_i_). Parasite density was measured every 24 h, with C_i_ being ∼10^6^ cells/ml. Parasite cultures were maintained until stabilisation of population doubling time was observed, with genomic and RNA samples collected at this point for comparative analysis against the first extraction.

### Microscopy

Light microscopy was carried out on live cells adhered to glass (Dean and Sunter, 2020). A sample of 0.5 ml culture in exponential growth was harvested by centrifugation (1200 ***g*** for 3 min) and washed three times with vPBS (Voorheis modified PBS; PBS supplemented with 10 mM glucose and 46 mM sucrose), before resuspension in ∼50 μl vPBS, from which a 5 μl sample was placed on a glass slide and the coverslip added. DNA was stained with Hoechst 33342 included in the first vPBS wash. Images were captured on Zeiss Imager Z2 microscope (Carl Zeiss, Jena, Germany) with a Plan-Apochromat 63× NA 1.4 oil objective and a Hamamatsu Flash 4 camera (Hamamatsu Photonics, Hamamatsu, Japan) using Zen 3.10 (Carl Zeiss, Jena, Germany).

For each tagged strain, three independent slides were prepared, and a minimum of ten images were acquired per slide. Kinetoplasts (K, mitochondrial DNA) and nuclei (N) were counted from micrographs to determine the cell cycle stage, with only G1-stage cells considered for quantitative localisation analysis. ESB1 foci (mNG, green channel) and RPA2, VEX1 or VEX2 foci (mCh, red channel) were identified. Once all green and red foci were mapped, number per cell and the distance between each ESB1 focus and its nearest neighbouring red focus was measured.

### Customized GFF and genome files

We previously generated a cell line-specific genome with 5′ and 3′ mRNA UTRs mapped using RNA-seq (European Nucleotide Archive, accession number PRJEB8829, Fiebig et al., 2015) for our laboratory strain of *Leishmania mexicana* MNYC/BZ/62/M379 expressing Cas9 and T7 RNA polymerase (Beneke et al., 2023), which was used as the basis of all analyses. This genome was published with gene IDs which begin with the prefix LmxM379c instead of LmxM and have a one-to-one correspondence. For reporting our results, for simplicity, we use the LmxM prefix.

### Transcriptomic analysis

Total RNA was extracted using the RNeasy Mini Kit (Qiagen) according to the manufacturer's protocol. RNA was eluted in 30 μl of nuclease-free water, stored at −80°C. For sequencing, mRNAs were enriched using poly-dT affinity, cDNA generated using random hexamer primers, and subject to short read paired end sequencing using BGI-SEQ 500 (paired end 100 bp reads, nominal insert size 200 bp). All were post-processed with Rcorrector (Song and Florea, 2015) and trimmed with TrimGalore (Krueger et al., https://github.com/felixkrueger/trimgalore; Zenodo, doi:10.5281/zenodo.5127899).

A reference genome for our parental clonal C9T7 cell line was generated by aligning genomic sequencing reads to our reference *L. mexicana* MNYC/BZ/62/M379 expressing Cas9 and T7 genome (Beneke et al., 2023) using BWA MEM2 (Vasimuddin et al., 2019) and generating a polished version of the genome using Pilon (Walker et al., 2014). Coordinates in general feature format (GFF) file describing the gene coordinates was adjusted based on insertions and deletions recorded in the variant call format (VCF) output from Pilon, to give an experiment-specific parental genome and genome annotations.

To quantify transcript abundance, the FASTQ reads were mapped to the corresponding reference transcriptome, which was derived from the customized GFF and genome files. A minimum mapping quality threshold (q) of 10 was applied, and only properly mapped reads (−F 0×04) were retained. The number of mapped reads per gene was determined using the idxstats command from the SAMtools library and those with fewer than 20 reads were removed from the downstream analysis. From these data, the Reads Per Kilobase per Million mapped reads (RPKM) were calculated for each replicate, and their standard deviation was determined. RPKM were then compared to the parental C9T7 RPKM using a one-sample Z test, then *P*-values plotted against log_2_ RPKM ratio (mutant RPKM/parental RPKM) for volcano plots.

For transcriptome-wide analysis of differentially expressed genes, these were filtered based on an absolute log_2_ ratio greater than 1 and −log_10_(*P*-values) exceeding 5.26 (*P*=5.45×10^–6^): a Bonferroni correction applied to the *P*-value threshold of 0.05, accounting for the 9182 genes annotated for *L. mexicana*. To identify candidate genes whose transcript abundance changed between the final and initial weeks, no multiple comparison correction to the threshold *P*-value of 0.05 was made as these were handled as individual candidates.

The pileup depth of RNA-seq reads aligned to the predicted mRNAs sequences of the deleted genes was assessed in both parental and deletion strains. Read depth values were grouped into bins (target length 100 bp, rounded based on transcript length) for comparative analysis. The average depth values of each bin in the knockout strains were then normalized against their corresponding values in the parental strain to ensure equal-scale representation.

### Amastin identification and classification

Genome annotation of the TriTrypDB *Leishmania mexicana* reference genome (strain MHOM/GT/2001/U1103) (Alvarez-Jarreta et al., 2024) from which our *L. mexicana* MNYC/BZ/62/M379 expressing Cas9 and T7 genome annotation was derived does not include consistent amastin and tuzin names. Consequently, we identified all amastin and tuzin genes by performing a BLASTp search using all named amastins and tuzins against the entire *Leishmania mexicana* predicted proteome, applying a threshold to exclude hits with an e-value less than 10^−5^. Some amastins have ‘partial’ in the gene ID and are fragments on unassembled genome fragments, which were excluded.

Amastin families were assigned by constructing a phylogeny of our gathered amastin sequences using the ‘build’ function of ETE3 3.1.3 (Huerta-Cepas et al., 2016) as implemented on GenomeNet (https://www.genome.jp/tools/ete/). Alignment was performed with MAFFT v6.861b with the default options (Katoh and Standley, 2013). The maximum likelihood tree was inferred using IQ-TREE 1.5.5 ran with ModelFinder and tree reconstruction (Nguyen et al., 2015). Best-fit model according to BIC was JTT+G4. Tree branches were tested by SH-like aLRT with 1000 replicates. Amastins were assigned to families by manual comparison of the previously published (de Paiva et al., 2015) and this new phylogenetic tree.

### Genomic analysis

To characterize genetic differences resulting from gene deletion and subsequent growth recovery, sequencing reads from genomic DNA were aligned to the corresponding parental strain genome using Burrows–Wheeler Aligner-MEM (BWA-MEM, v0.7.17-r1188; Li, 2023 preprint, https://github.com/lh3/BWA) with default parameters. The aligned reads were then sorted and indexed using SAMtools.

Genomic variants were identified using FreeBayes (Garrison and Marth, 2012 preprint), a Bayesian genetic variant caller designed to detect single-nucleotide polymorphisms (SNPs), insertions, deletions and complex variants. Newly generated (first week) LmxESB1 and LmxFAZ5 mutants were compared to the parental C9T7 genome. LmxESB1 and LmxFAZ5 genome changes during growth rate recovery were identified by comparison to the first week sample from the same clone.

Variant annotation was performed with SnpEff and SnpSift. First, a database was built for each reference genome using the genome FASTA file, along with the corresponding CDS and proteome FASTA files. SnpEff was then used for annotation, followed by SnpSift-based filtering to refine variants based on quality scores and other annotations in the VCF file. Only SNPs and single InDels within coding sequences (CDS) were considered, only considering variants with a quality of score of 30 or higher. Finally, variants which occurred across independent clones were mapped, and their potential phenotypic implications were manually assessed.

### Study design

Sample size is reported in the figure legends. All experiments were performed three times unless specified in the figure legends. No data were excluded from the analyses. All analysis was performed by researchers aware of experimental designation and no randomisation occurred during these studies.

## Supplementary Material



10.1242/joces.264459_sup1 Supplementary information

Table S1. ESB1KO_P_IW

Table S2. FAZ5 KO

Table S3. ESB1KO_P_FW_IW_FW

Table S4. DRBD13 KO

Table S5. RBP10 KO

Table S6. NIFP1 KO

Table S7. Primers for N/C terminal tagging, gene knockout, and integration confirmation
