# Supplementary material for: Regulation of Leishmania surface coat proteins by the nuclear protein ESB1
Source: J Cell Sci. 2026 Jul 13;139(13):jcs264459. doi: 10.1242/jcs.264459 (PMC13405223; doi:10.1242/jcs.264459)
Supplement: Supplementary information [file joces-139-264459-s1.pdf]

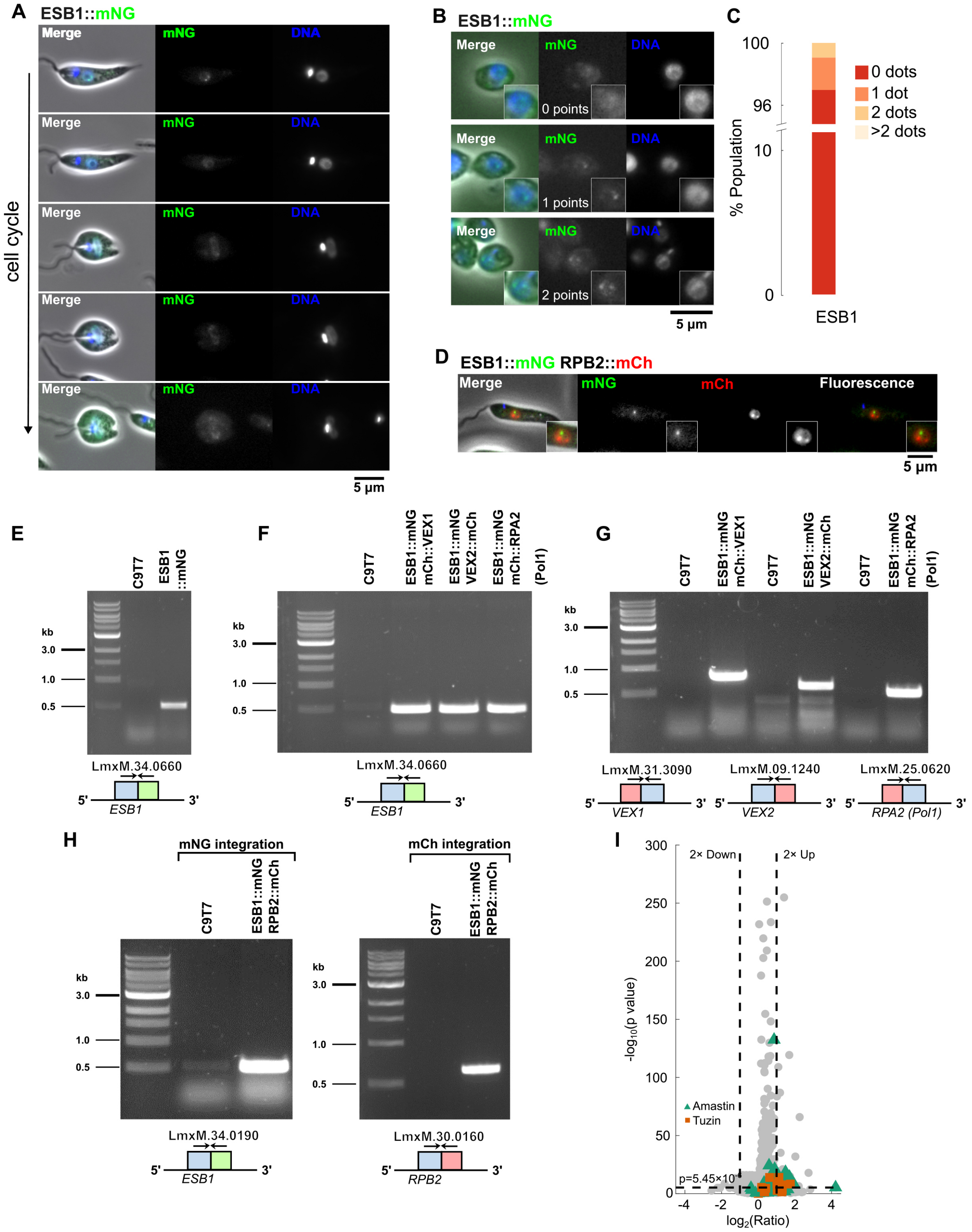

Journal of Cell Science • Supplementary information

Fig. S1. Additional ESB1 localisation analysis and integration confirmation. A. Subcellular localisation of LmxESB1 during the cell cycle. **B.** Subcellular localisation of LmxESB1 in axenic amastigotes. Representative fluorescence micrographs of amastigotes expressing ESB1::mNG showing typical cells with 0 bright foci, and occasional cells with 1, 2 or more bright fluorescence foci. Separate mNG and DNA fluorescence images are shown, along with an overlay with phase contrast. A detailed view of the nucleus is inset. **C.** The proportion of cells with different numbers of bright nuclear foci shown on the right (n = 150 cells from one biological repeat). **D.** Position of ESB1 foci relative to RNA polymerase II (RPB2 subunit). Representative fluorescence micrographs of promastigotes expressing ESB1::mNG and RPB2::mCh showing cells with one or more foci of both tagged proteins. Separate mNG and mCherry fluorescence images are shown, along with an overlay of all fluorescence images, including DNA stain, and an overlay with phase contrast. A detailed view of the nucleus is inset. **E-H.** PCR confirmation of the correct genomic integration of mNG and mCh tags. **E.** Integration of mNG at the 3’ end of ESB1 in the gDNA of the ESB1::mNG cell line. **F.** Integration of mNG at the 3’ end of ESB1 in the gDNA of cells expressing ESB1::mNG with VEX1, VEX2, or RPA2 tagged with mCh. **G.** Integration of mCh at the expected end of the gene in the gDNA of cells expressing ESB1::mNG with VEX1, VEX2, or RPA2 tagged with mCh. **H.** Integration of mNG at the 3’ end of ESB1 and mCh at the 3’ end of RPB2 in the gDNA of the cells expressing ESB1::mNG with RPB2::mCh. **I.** Plot of the ratio of transcript abundance in the parental cell line to the LmxESB1 deletion mutant, against statistical significance of abundance change. Each data point represents a single transcript, average of n = 3 clonal deletion populations The horizontal dashed line represents p = 0.05 with Bonferroni multiple comparison correction ($p = 5.45 \times 10^{-6}$, considering 9,182 total hypotheses), the vertical dashed lines represent two-fold increased or decreased transcript abundance. Amastin and Tuzin genes are highlighted.

Journal of Cell Science • Supplementary information

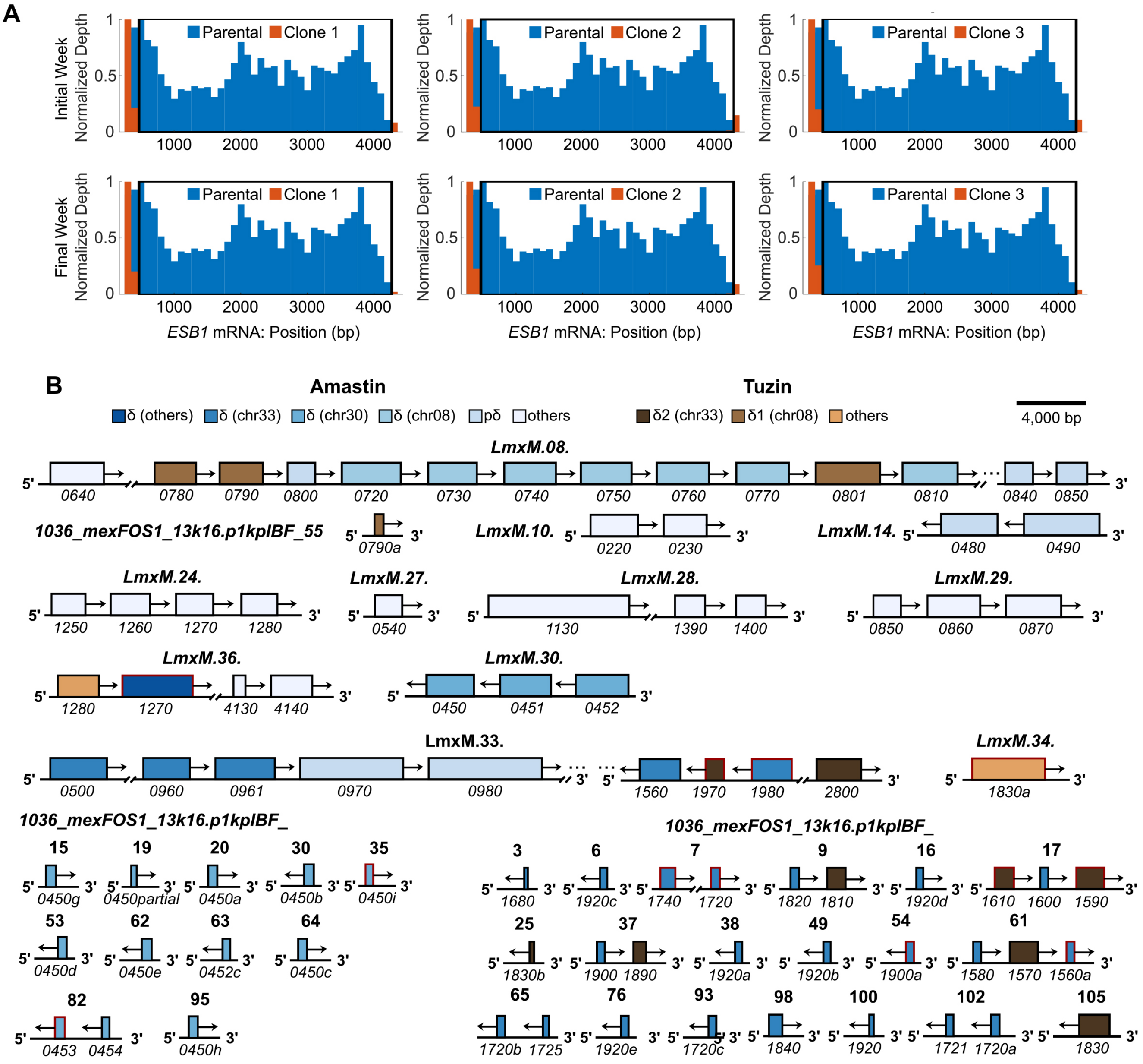

Fig. S2. ESB1 deletion confirmation and amastin and tuzin chromosomal locations. A. Normalised read depth aligning RNAseq data to the *LmxESB1* gene for each deletion mutant clone (columns: Clone 1, 2 and 3) at the initial and final week (rows) in comparison to the parental cell line. Black box represents the bounds of the CDS within the mRNA. **B.** Genomic position of amastin and tuzin genes in the *L. mexicana* genome. Many are truncated and present on unassembled contigs.

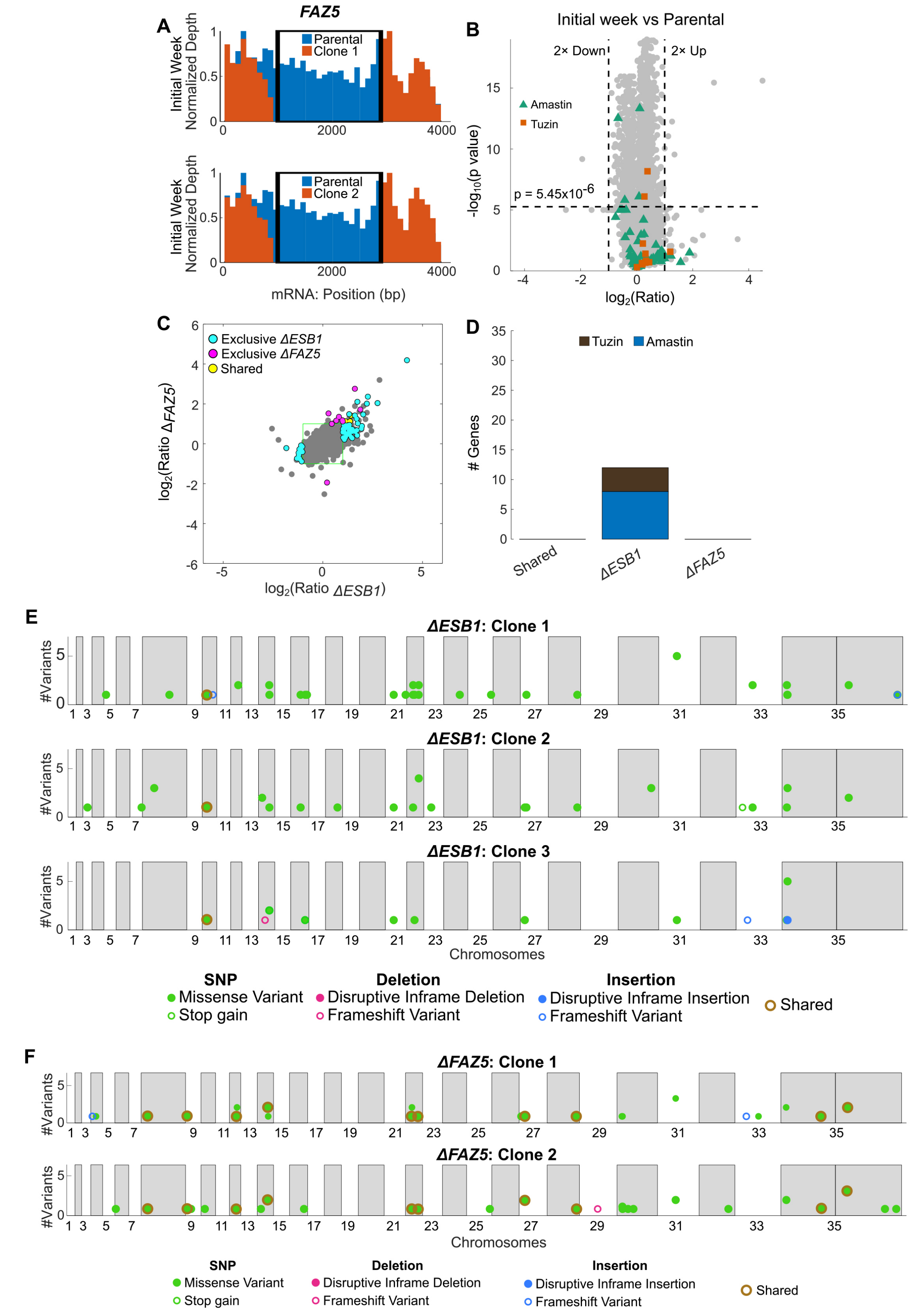

Fig. S3. The *Leishmania* genome is stable upon deletion of ESB1 and control genes. A. Normalised read depth aligning RNAseq data to the *Lmx*FAZ5 mRNA for each deletion mutant clone in comparison to the parental cell line. Black box represents the bounds of the CDS within the mRNA. **B.** Plot of the ratio of transcript

Journal of Cell Science • Supplementary information

abundance in the parental cell line to the LmxFAZ5 deletion mutants, against statistical significance of abundance change. Each data point represents a single transcript, average of n = 2 clonal deletion populations. The horizontal dashed line represents $p = 0.05$ with Bonferroni multiple comparison correction ($p = 5.45 \times 10^{-6}$, considering 9,182 total hypotheses), the vertical dashed lines represent two-fold increased or decreased transcript abundance. Amastin and Tuzin genes are highlighted. **C.** Correlation of the fold change of transcript abundance following LmxFAZ5 deletion in comparison to the parental cell line, plotted against fold change of transcript abundance following LmxESB1 deletion compared to the parental cell line. Each data point represents the mean from the clonal deletion mutant populations. Transcripts statistically significantly up or downregulated at least twofold are highlighted, colour coded by whether they have altered expression in one or both deletion mutant clonal populations. The green box represents less than two-fold increase or decrease. Cyan, magenta and yellow data points represent transcript significantly changed in only LmxESB1 deletion relative to parental, the other deleted gene relative to parental, or both, respectively. **D.** Number of significantly upregulated amastin transcripts from D, categorised by change in one or both mutant cell lines. **E.** Analysis of the genome of the three ΔLmxESB1 clones, mapping detected insertion and deletion mutations and single nucleotide polymorphisms (SNPs) when causing a missense or stop codon. Horizontal axis represents position in the genome, alternating gray boxes represent chromosome bounds. Only protein coding sequences were analysed. Shared represents mutations found in all three clones. **F.** Analysis of the genome of the two ΔLmxFAZ5 clones, mapping detected insertion and deletion mutations and single nucleotide polymorphisms (SNPs) when causing a missense or stop codon. Horizontal axis represents position in the genome, alternating gray boxes represent chromosome bounds. Only protein coding sequences were analysed. Shared represents mutations found in both clones.

Journal of Cell Science • Supplementary information

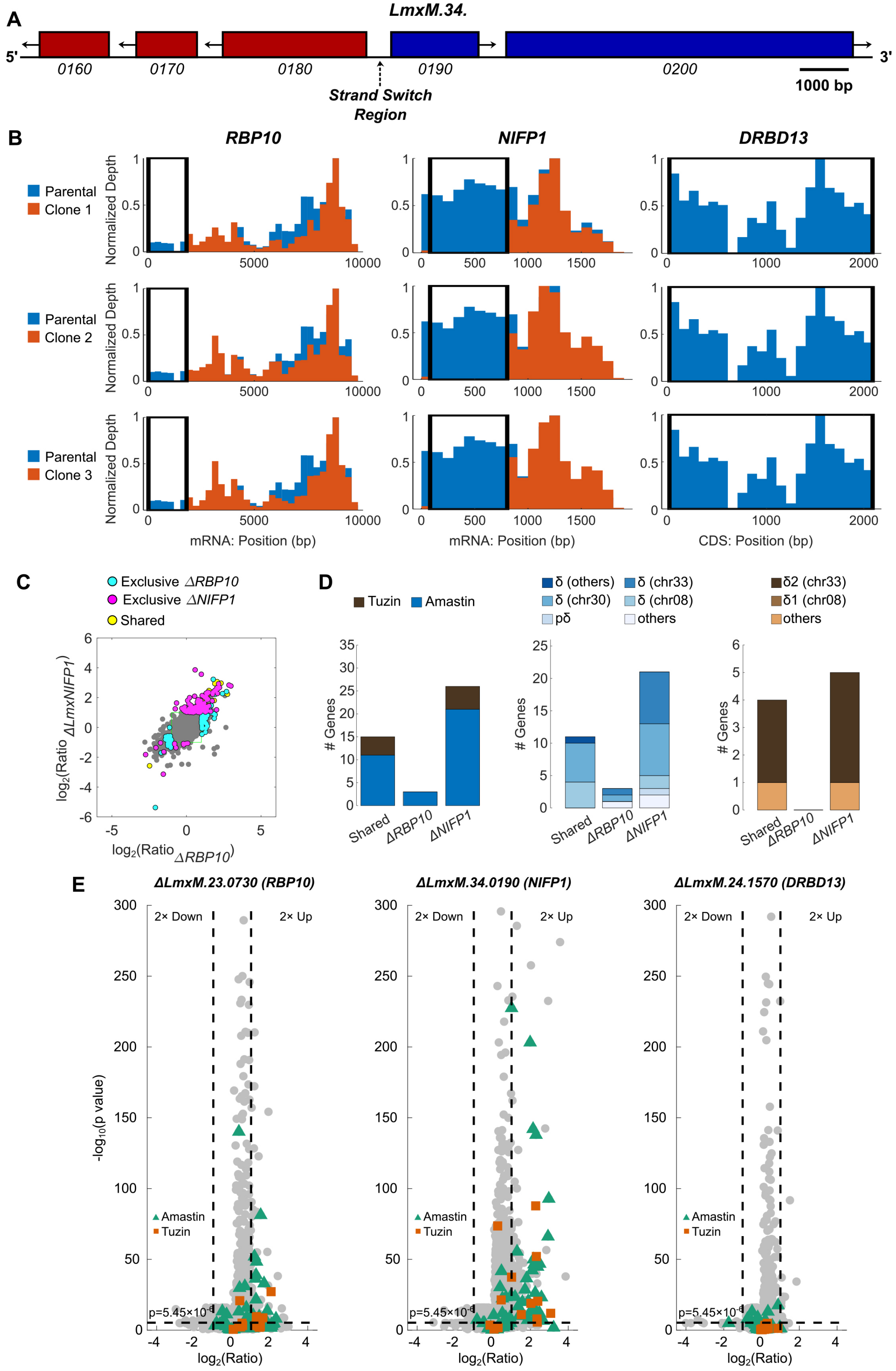

Journal of Cell Science • Supplementary information

Fig. S4. Additional analysis of the RBP10, NIFP1 and DRBD13 deletion mutants. A. Diagram of the genomic locus of NIFP1, immediately downstream of a strand-switch region. **B.** Read depth aligning RNAseq data to the RBP10 (LmxM.23.0730), NIFP1 (LmxM.34.0190) and DRBD13 (LmxM.24.1570) mRNAs for each deletion mutant clone in comparison to the parental cell line. Read depth values were normalized against parent level. Black box represents the bounds of the CDS within the mRNA. The bounds of the 5′ and 3′ UTR have not been mapped for DRBD13, so only the CDS is plotted. **C.** Correlation of the fold change of transcript abundance following RBP10 deletion in comparison to the parental cell line, plotted against fold change of transcript abundance following NIFP1 deletion compared to the parental cell line. Each data point represents the mean from n = 3 clonal deletion mutant populations. Transcripts statistically significantly up or downregulated at least twofold are highlighted, colour coded by whether they have altered expression in one or both deletion mutant clonal populations. The green box represents less than two-fold increase or decrease. Cyan, magenta and yellow data points represent transcript significantly changed ($p <= 0.5$) in only ESB1 deletion relative to parental, the other deleted gene relative to parental, or both, respectively. **D.** Number of significantly upregulated amastin transcripts from D, categorised by change in one or both mutant cell lines, broken down by type. Left: for δ-amastins, and which chromosome (chr) they are encoded on, proto-δ-amastins (pδ) and other (amastin families α, β or γ). Right: Number of significantly upregulated tuzin transcripts from A, broken down by which chromosome they are encoded on. **E.** Plot of the ratio of transcript abundance in the parental cell line to the RBP10, NIFP1, and DRBD13 deletion mutants, against statistical significance of abundance change. Each data point represents a single transcript, average of n = 3 clonal deletion populations The horizontal dashed line represents $p = 0.05$ with Bonferroni multiple comparison correction ($p = 5.45 \times 10^{-6}$, considering 9,182 total hypotheses), the vertical dashed lines represent two-fold increased or decreased transcript abundance. Amastin and Tuzin genes are highlighted.

Journal of Cell Science • Supplementary information

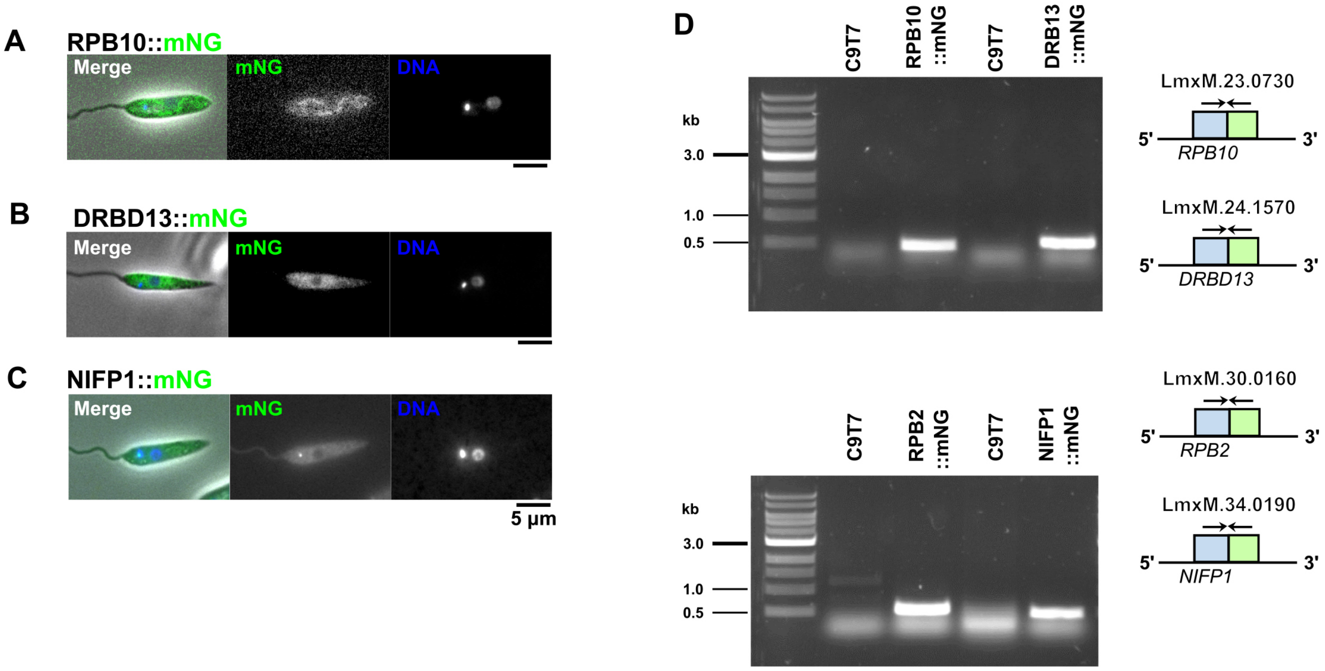

Fig. S5. Localisation of RBP10, DRBD13, and NIFP1. A. Subcellular localisation of RBP10 in promastigotes. Representative fluorescence micrographs of promastigotes expressing RBP10::mNG. **B.** Subcellular localisation of DRBD13 in promastigotes. Representative fluorescence micrographs of promastigotes expressing DRBD13::mNG. **C.** Subcellular localisation of NIFP1 in promastigotes. Representative fluorescence micrographs of promastigotes expressing NIFP1::mNG. **D.** PCR confirmation of the correct genomic integration of mNG tags. NB images of RBP2 are presented in Figure S1.

Journal of Cell Science • Supplementary information

Table S1. ESB1KO_P_IW

Available for download at
https://journals.biologists.com/jcs/article-lookup/doi/10.1242/jcs.264459#supplementary-data

Table S2. FAZ5 KO

Available for download at
https://journals.biologists.com/jcs/article-lookup/doi/10.1242/jcs.264459#supplementary-data

Table S3. ESB1KO_P_FW_IW_FW

Available for download at
https://journals.biologists.com/jcs/article-lookup/doi/10.1242/jcs.264459#supplementary-data

Table S4. DRBD13 KO

Available for download at
https://journals.biologists.com/jcs/article-lookup/doi/10.1242/jcs.264459#supplementary-data

Table S5. RBP10 KO

Available for download at
https://journals.biologists.com/jcs/article-lookup/doi/10.1242/jcs.264459#supplementary-data

Table S6. NIFP1 KO

Available for download at
https://journals.biologists.com/jcs/article-lookup/doi/10.1242/jcs.264459#supplementary-data

Table S7. Primers for N/C terminal tagging, gene knockout, and integration confirmation

Available for download at
https://journals.biologists.com/jcs/article-lookup/doi/10.1242/jcs.264459#supplementary-data
