# Supplementary material for: Regulation of Leishmania surface coat proteins by the nuclear protein ESB1
Source: J Cell Sci. 2026 Jul 13;139(13):jcs264459. doi: 10.1242/jcs.264459 (PMC13405223; doi:10.1242/jcs.264459)
Supplement: Supplementary file 1 [file joces-139-264459_review_history.pdf]

Journal of Cell Science

Regulation of *Leishmania* surface coat proteins by the nuclear protein ESB1

Jorge Adrián Arias del Angel, Ulrich Dobramysl, Laura Davidson, Richard J. Wheeler and Jack D. Sunter
DOI: 10.1242/jcs.264459

Editor: Megan King

Review timeline
Original submission: 17 September 2025
Editorial decision: 3 December 2025
First revision received: 2 April 2026
Editorial decision: 5 May 2026
Second revision received: 27 May 2026
Accepted: 3 June 2026

Original submission

First decision letter

MS ID#: jcs.264459

MS TITLE: Regulation of surface coat proteins by a life stage-specific Leishmania nuclear body

AUTHORS: Jorge Adrián Arias del Angel; Ulrich Dobramysl; Richard J Wheeler; Jack D Sunter
ARTICLE TYPE: Research Article

Dear Dr. Sunter,

We have now reached a decision on the above manuscript.

As you will see, while Reviewer 1 has only minor comments to be addressed on revision, Reviewer 2 raises a number of more substantial criticisms that prevent me from accepting the paper at this stage. They suggest, however, that a revised version might prove acceptable, if you can address their concerns. If you think that you can deal satisfactorily with the criticisms on revision, I would be pleased to see a revised manuscript. We would then return it to the reviewers.

Reviewer 1

SUMMARY OF THE ADVANCE MADE IN THIS PAPER AND ITS POTENTIAL SIGNIFICANCE TO THE FIELD

In this manuscript, the authors document the existence of a microscopically visible nuclear structure in which the L. mexicana EBS1 orthologue is localised. In previous articles, this group has contributed relevant information about T. brucei EBS1 and its participation in conforming the nuclear body responsible for VSG expression in this parasite. It is the first time that a similar structure has been documented in Leishmania. However, the L. mexicana nuclear body does not contain RNApol1 (and other proteins found in the T. brucei nuclear body), as no colocalization was found in the immunofluorescence images shown in Figure 1. Afterwards, the authors deleted the L. mexicana EBS1-coding gene and, remarkably, the mRNAs coding for a group of surface proteins (amastins) increased, suggesting that this nuclear body is engaged in regulating the expression of

© 2026. Published by The Company of Biologists under the terms of the Creative Commons Attribution License (https://creativecommons.org/licenses/by/4.0/).

this particular set of genes. The manuscript contains additional experiments, in which further deletion lines were created and analysed by RNA-seq. Overall, this work contains a huge amount of data and represents a brilliant contribution that opens a new perspective in addressing mechanisms of gene expression regulation in this parasite.

SUGGESTIONS TO AUTHORS

Major comments [Please request additional experiments only if they are essential for supporting the conclusions; authors should be encouraged to highlight any claims that are preliminary or speculative, or to discuss any pitfalls or alternative interpretations in a 'Limitations' section]

Minor comments

There are a few minor points that the authors should consider during the manuscript revision.

1. LmxM.34.0190 (NIFP1). In UniProt, the recommended name for this protein is Mitochondrial import inner membrane translocase subunit TIM50. However, in agreement with a previous publication, this protein is described here as a phosphatase. Could the authors comment on these features? Do the authors have any clue about the location of this protein?

2. Page 3 (Line 53). It should be read: RBP10.

3. Page 13 (Line 39). Please, check if LmxM.24.1570 correspond to DRBD13 or DRBD12 (PMID: 16339728)

4. Page 14. To determine the doubling time of promastigotes, the authors measured cell density. How was cell density determined? Please, add this information.

5. Page 15 (Lines 13-15). Authors have generated "an experiment-specific parental genome and genome annotations". Is this information available anywhere?

6. Page 15 (Line 19). Again, is the reference transcriptome available anywhere?

7. Page 4 (lines 34-35). The authors conclude that LmxESB1 does not co-localise with RNApol1, and the microscope images support this conclusion. Has it been checked whether ESB1 might be interacting with RNApol2?

8. Page 5 (Line 50-51) Could the authors provide a supplementary table listing the ID of those transcripts that were significantly up- or down-regulated because of ESB1 deletion? The same would be appropriate for the transcripts whose levels resulted altered after the deletion of NIFP1 or RBP10.

9. In legend to figure S1, it is not indicated what the difference is between the three images (either for the initial or the final week) shown in panel C.

10. In legend S3. Please, add the ID for the gene coding for LmxNIFP1.

11. According to panel B of Figure S3, the 3'-UTR of RBP10 is quite large. How was this region determined?

Reviewer 2

SUMMARY OF THE ADVANCE MADE IN THIS PAPER AND ITS POTENTIAL SIGNIFICANCE TO THE FIELD

In this study, the protein with closest homology to Trypanosoma brucei ESB1 has been investigated in Leishmania mexicana. The authors suggest that ESB1 does have a necessary role in surface protein expression in L. mexicana, yet in stark contrast to T. brucei, it is expressed in promastigote stages and acts as a negative (as opposed to positive in T. brucei) regulator of surface protein expression. Although of interest to the trypanosome/leishmania field, the function of this protein is

© 2026. Published by The Company of Biologists under the terms of the Creative Commons Attribution License (https://creativecommons.org/licenses/by/4.0/).

not yet clear. The authors focus on specific transcriptional changes, and some data are not provided. Given the elaborate mechanisms of surface protein expression that may be unique to T. brucei, it is not far-fetched that ESB1 homologs may have a seemingly opposing function in L. mexicana. The authors cannot provide mechanistic insight into LmxESB1 function currently but report expression pattern and a change in transcriptome after deletion.

SUGGESTIONS TO AUTHORS

Major comments

Several figures and data are missing:

1. Figure S1D is referred to but not presented.
2. The authors have only included G1 (1N1K) cells in their analysis when counting ESB1 foci - why? What does this protein look like in other stages of the cell cycle? This selection seems unnecessary.
3. Did the authors validate any of the tagged cell lines? Is the tag integrated in the correct region and is the protein the expected size?
4. Figures S2B-E are not included in the manuscript as described in the text.
5. Page 6, line 34 - Fig 3B does not match the data referred to.
6. Page 6, line 40 - Fig 3A is not a growth curve, and growth curves are not provided.
7. The results of the transcriptomics analysis for all knockout cell lines are not provided full.

Overall, the data shown do not provide a mechanism for the proposed ESB1 function in amastin repression and does not fully investigate alternative roles for ESB1 that could explain its conservation outside of T. brucei, leaving several gaps to the claims.

* The title "Regulation of surface coat proteins by a life stage-specific Leishmania nuclear body" - This work does falls short of this title as it does not currently describe a nuclear body, or absolute stage specificity, only the localisation of EBS1, and so this title should be reconsidered.
* "LmxESB1 localises to a promastigote-specific nuclear body." Yet it is seen in some amastigote cells (albeit fewer), so isn't quite accurate. Additionally, only G1 cells are examined - why? Are both alleles tagged? Is the tag confirmed by PCR and/or western blot? Is ESB1 expressed in any of other cell cycle phases of either.
* "Its deletion caused derepression of Î´-amastin expression from a specific chromosomal locus." Yet amastins and genes from other loci are also derepressed, and others are downregulated. Given that ~5/8 of the amastin genes altered by ESB1 are on chromosome 33 (judging by figure 3), this is not a strong enrichment on which to argue specificity, so why limit the descriptions to just this locus. More experiments are required to prove this if true, e.g., localisation of ESB1 to this locus on chromosome 33 or interactions of ESB1 with other proteins to ensure repression of this specific locus.
* The authors focus on amastin and tuzin, which are of interest due to the role of ESB1 in T. brucei surface protein expression. However, other genes are altered - what are the 45 and 16 genes that show changed regulation, and how many of them are amastin and tuzin? This is not clear from the figures/text alone. The full results from the various transcriptomic analyses need to be provided.
* The paper does not fully explore other possible roles for ESB1, to either rule them in or out. For example, the top gene with the highest fold change in the ESB1 deletion cell line is LmxM.34.0070 (deletion based on Fig 2D), a putative prohibitin, and this persistence with prolonged culture. ESB1 thus seems to have an important role in this genes repression but this is not explored?
* Similarly, looking at the genome analysis figure there is a shared mutation on chromosome 9 for all the ESB1 knockout clones (Fig S2)? what is it?
* The claim that two proteins, RBP10 and NIFP1, are responsible for recovery of the ESB1 knockout lines, as they act in parallel with ESB1 to repress amastin and tuzins, could be convincing with additional experiments but currently isn't clear. RBP10 and NIFP1 can be tagged and localised, protein interactions established for example.
* Similarly on Page 6, line 43: "Analysis of the deletion mutant transcriptomes showed that the slow growth of LmxRBP10 and LmxNIFP1 deletion mutants both correlated with an increase of amastin and tuzin transcripts" - no such growth data are provided and are there any genes with altered expression in all mutants? (other than the amastins and tuzin).

© 2026. Published by The Company of Biologists under the terms of the Creative Commons Attribution License (https://creativecommons.org/licenses/by/4.0/).

Minor comments

1. Fig 2D and E: The legend states the amastin genes are highlighted in blue but do not appear to be; equally, the RNA regulators chosen for investigation are not underlined.
2. In Fig 3, the numbers of amastin and tuzin genes altered do not appear to match between A and C/D - was a different significance threshold used? Why?
3. What % of the amastin genes in these regions (on chromosomes 33, 3, and 8) are altered? How would RBP10 act on specific chromosome regions? What does RBP10 localisation look like? Very few shared.
4. Page 7, lines 45-48 - typos in this sentence? The meaning is unclear.
5. Typo on page 13, line 18 - pPLOT.

First revision

Author response to reviewers' comments

Comments from the Reviewers:

Reviewer 1: SUMMARY OF THE ADVANCE MADE IN THIS PAPER AND ITS POTENTIAL SIGNIFICANCE TO THE FIELD

In this manuscript, the authors document the existence of a microscopically visible nuclear structure in which the L. mexicana EBS1 orthologue is localised. In previous articles, this group has contributed relevant information about T. brucei EBS1 and its participation in conforming the nuclear body responsible for VSG expression in this parasite. It is the first time that a similar structure has been documented in Leishmania. However, the L. mexicana nuclear body does not contain RNApol1 (and other proteins found in the T. brucei nuclear body), as no colocalization was found in the immunofluorescence images shown in Figure 1. Afterwards, the authors deleted the L. mexicana EBS1-coding gene and, remarkably, the mRNAs coding for a group of surface proteins (amastins) increased, suggesting that this nuclear body is engaged in regulating the expression of this particular set of genes. The manuscript contains additional experiments, in which further deletion lines were created and analysed by RNA-seq. Overall, this work contains a huge amount of data and represents a brilliant contribution that opens a new perspective in addressing mechanisms of gene expression regulation in this parasite.

Thank you very much for this enthusiastic positive review. We are very excited by our discoveries and were very pleased to see this recognised as a “brilliant contribution” of importance going forward as it “opens a new perspective in addressing mechanisms of gene expression regulation in this parasite”.

SUGGESTIONS TO AUTHORS

Minor comments

There are a few minor points that the authors should consider during the manuscript revision.

1. LmxM.34.0190 (NIFP1). In UniProt, the recommended name for this protein is Mitochondrial import inner membrane translocase subunit TIM50. However, in agreement with a previous publication, this protein is described here as a phosphatase. Could the authors comment on these features? Do the authors have any clue about the location of this protein?

The corresponding UniProt entry (E9B5Q7) is indeed annotated TIM50, and we did not previously see this. However, the actual TIM50 protein in L. mexicana is LmxM.26.0160, based on orthology with the annotated TIM50 in T. brucei in TriTrypDB. The confusion comes from the NIF FCP1 homology domain - TIM50 and NIFP1 both have this domain, but TIM50 has a large N terminal domain missing from NIFP1.

© 2026. Published by The Company of Biologists under the terms of the Creative Commons Attribution License (https://creativecommons.org/licenses/by/4.0/).

We have now endogenously tagged NIFP1 and the fusion protein localises to the cytoplasm (Figure S5C). This matches the localisation of the T. brucei ortholog, which is also cytoplasmic, see TrypTag.org/?id=Tb927.10.4180.

2. Page 3 (Line 53). It should be read: RBP10.

We have ensured throughout the manuscript that RBP10 is referred to correctly.

3. Page 13 (Line 39). Please, check if LmxM.24.1570 correspond to DRBD13 or DRBD12 (PMID: 16339728)
We have carefully checked: The ortholog in T. brucei is Tb927.8.6650 which is listed as DRBD13 in the reference you have provided.

4. Page 14. To determine the doubling time of promastigotes, the authors measured cell density. How was cell density determined? Please, add this information.

The cell density was measured using a Beckman Coulter Counter and we have now added this detail to the methods. These cell densities were then used to calculate the doubling time and this approach was already in the manuscript: “doubling time was then calculated by $t \times \log_{10}(2) / \log_{10}(C_f / C_i)$”

5. Page 15 (Lines 13-15). Authors have generated "an experiment-specific parental genome and genome annotations". Is this information available anywhere?

The master genome and genome annotation for this analysis is the *Leishmania mexicana* MNYC/BZ/62/M379 expressing Cas9 and T7 RNA polymerase doi.org/10.12688/wellcomeopenres.18575.1. The ‘experiment-specific parental genome’ refers to a polish of this genome with genome sequencing data from the identical cell line, as used for the parental line of the experiment. This is now available as supplementary data 1.

6. Page 15 (Line 19). Again, is the reference transcriptome available anywhere?

The genome fasta, genome annotation gff and transcriptome fasta for the strain-specific genome are available at the Zenodo repository linked with the above-mentioned paper zenodo.org/records/14621467. The experiment-specific genome has a handful of minor differences, i.e. SNPs and small indels, but otherwise the same.

7. Page 4 (lines 34-35). The authors conclude that LmxESB1 does not co-localise with RNApol1, and the microscope images support this conclusion. Has it been checked whether ESB1 might be interacting with RNApol2?

We have now endogenously tagged a subunit of the RNA Polymerase II machinery (RPB2 - LmxM.30.0160) with mCherry on top of a cell line already expressing LmxESB1 fused to mNG. In these cells RBP2 localises to one or two foci within the nucleus; however, these foci do not overlap with the LmxESB1::mNG foci and so it is unlikely these proteins are directly interacting (Figure S1D).

8. Page 5 (Line 50-51) Could the authors provide a supplementary table listing the ID of those transcripts that were significantly up- or down-regulated because of ESB1 deletion? The same would be appropriate for the transcripts whose levels resulted altered after the deletion of NIFP1 or RBP10.

We have now included the transcriptomic analysis of the different deletion cells as supplementary tables (Tables S1-6), with a tab for those transcripts significantly up- or down-regulated.

9. In legend to figure S1, it is not indicated what the difference is between the three images (either for the initial or the final week) shown in panel C.

© 2026. Published by The Company of Biologists under the terms of the Creative Commons Attribution License (https://creativecommons.org/licenses/by/4.0/).

We have now clarified this legend as follows - “Normalised read depth aligning RNAseq data to the *LmxESB1* mRNA for each deletion mutant clone (columns: Clone 1,2 and 3) at the initial and final week (rows)…” Please note this is now Figure S2A.

10. In legend S3. Please, add the ID for the gene coding for LmxNIFP1.

We have now included the gene IDs for RBP10, DRBD13, and NIFP1 in this figure legend.

11. According to panel B of Figure S3, the 3'-UTR of RBP10 is quite large. How was this region determined?

As described in the corresponding genome paper (doi.org/10.12688/wellcomeopenres.18575.1), 3’ UTRs were mapped using the most-commonly used spliced leader acceptor site upstream of the CDS and prior to the next annotated CDS, using the spliced leader and polyadenylation sites described in doi.org/10.1371/journal.ppat.1005186. A BLASTx search of the long upstream intergenic sequence against all trypanosomatid proteins gives no good hits, meaning it is unlikely to contain an unannotated CDS and is plausibly a long 3’ UTR. However, there are two runs of Ns in the genome sequence indicating assembly challenges. Manual inspection of the number of times the spliced leader acceptor site was observed suggests that a site was confidently detected, but may be for a cryptic intervening gene due to the assembly problems.

Reviewer 2: SUMMARY OF THE ADVANCE MADE IN THIS PAPER AND ITS POTENTIAL SIGNIFICANCE TO THE FIELD

In this study, the protein with closest homology to Trypanosoma brucei ESB1 has been investigated in Leishmania mexicana. The authors suggest that ESB1 does have a necessary role in surface protein expression in L. mexicana, yet in stark contrast to T. brucei, it is expressed in promastigote stages and acts as a negative (as opposed to positive in T. brucei) regulator of surface protein expression. Although of interest to the trypanosome/leishmania field, the function of this protein is not yet clear. The authors focus on specific transcriptional changes, and some data are not provided. Given the elaborate mechanisms of surface protein expression that may be unique to T. brucei, it is not far-fetched that ESB1 homologs may have a seemingly opposing function in L. mexicana. The authors cannot provide mechanistic insight into LmxESB1 function currently but report expression pattern and a change in transcriptome after deletion.

SUGGESTIONS TO AUTHORS

Major comments

Several figures and data are missing:

1. Figure S1D is referred to but not presented.

We apologise for the missing figure panels and have now ensured all those referred to in the manuscript are present. Note this is now Figure S2B.

2. The authors have only included G1 (1N1K) cells in their analysis when counting ESB1 foci - why? What does this protein look like in other stages of the cell cycle? This selection seems unnecessary.

In L. mexicana cell cultures, the vast majority of cells are 1K1N and hence we focused on that cell cycle stage. We have now revisited our images and inspected the localisation of ESB1 in cells at different points in the cell cycle and have now provided example images of these cells in Figure S1A. Given the rarity of cells in the latter stages of the cell cycle, we were unable to provide a statistical analysis of the localisation to determine if there were changes in ESB1 localisation associated with the cell cycle. However, in these cells there is a similar pattern of ESB1 localisation as seen in G1 cells, with ESB1 localised to the nucleus, with foci also seen.

3. Did the authors validate any of the tagged cell lines? Is the tag integrated in the correct region and is the protein the expected size?

© 2026. Published by The Company of Biologists under the terms of the Creative Commons Attribution License (https://creativecommons.org/licenses/by/4.0/).

To validate these tagged cell lines, we have performed PCR analysis on gDNA to ensure that the gene encoding the fluorescent protein is integrated at the correct position. Primers were designed that flanked the integration site and would generate a band of ~500 bp. This analysis is the supplementary figures (S1E-H; S5D) and showed that the gene encoding the fluorescent protein was fused to the open reading frame as expected.

4. Figures S2B-E are not included in the manuscript as described in the text.

We have now ensured that the presentation of the figures aligns with the description in the text. Note that due to addition of supplementary figures their numbering has changed.

5. Page 6, line 34 - Fig 3B does not match the data referred to.

We have now ensured that the text aligns with the figures.

6. Page 6, line 40 - Fig 3A is not a growth curve, and growth curves are not provided.

We have now included this growth curve data and apologise for its absence in the initial submission.

7. The results of the transcriptomics analysis for all knockout cell lines are not provided full.

Overall, the data shown do not provide a mechanism for the proposed ESB1 function in amastin repression and does not fully investigate alternative roles for ESB1 that could explain its conservation outside of T. brucei, leaving several gaps to the claims.

We have now included the analysis of the transcriptomic data in supplementary tables (Table S1-6). In addition, we have performed additional experiments, modified the text, presenting our results with more detail and have included caveats and alternative explanations to the discussion. While we acknowledge that we have not provided a specific mechanism for LmxESB1, we have constrained the possible mechanisms, and we believe that determining a full mechanism is beyond the scope of the manuscript. We have included details in the discussion of the next steps required to do this. Please see below for responses to specific comments.

* The title "Regulation of surface coat proteins by a life stage-specific Leishmania nuclear body" - This work does falls short of this title as it does not currently describe a nuclear body, or absolute stage specificity, only the localisation of EBS1, and so this title should be reconsidered.

We have changed the title to “Regulation of Leishmania surface coat proteins by the nuclear protein ESB1” to more accurately reflect our results.

* "LmxESB1 localises to a promastigote-specific nuclear body." Yet it is seen in some amastigote cells (albeit fewer), so isn't quite accurate. Additionally, only G1 cells are examined - why? Are both alleles tagged? Is the tag confirmed by PCR and/or western blot? Is ESB1 expressed in any of other cell cycle phases of either.

Regarding the first point raised by the reviewer, the decrease is from a nuclear focus visible in ~40% of promastigotes to ~3% of axenic amastigotes. We argue that this is a very convincing decrease, especially as the axenic amastigotes are recently differentiated from promastigotes (72h). This suggests that this is promastigote-specific body.

Only a single allele of LmxESB1 was tagged with mNG, which is the approach used routinely in the field when determining protein localisation by fusing to a fluorescent protein. Moreover, we have confirmed that the gene encoding the mNG tag is correctly integrated into the genome.

We have now done additional analysis of the promastigote cells expressing LmxESB1 fused to mNG and shown that it is expressed throughout the cell cycle and localises to the nucleus, with additional foci seen in cells (Figure S1A). In our hands, due to lower growth rate we see very few axenic amastigotes in other cell cycle stages beyond G1. Combined with the very infrequent nuclear signal, we have been unable to confirm the localisation of LmxESB1 during the amastigote

© 2026. Published by The Company of Biologists under the terms of the Creative Commons Attribution License (https://creativecommons.org/licenses/by/4.0/).

cell cycle; however, we have no reason to believe that the LmxESB1 localisation is regulated in a cell cycle manner.

* "Its deletion caused derepression of δ-amastin expression from a specific chromosomal locus." Yet amastins and genes from other loci are also derepressed, and others are downregulated. Given that ~5/8 of the amastin genes altered by ESB1 are on chromosome 33 (judging by figure 3), this is not a strong enrichment on which to argue specificity, so why limit the descriptions to just this locus. More experiments are required to prove this if true, e.g., localisation of ESB1 to this locus on chromosome 33 or interactions of ESB1 with other proteins to ensure repression of this specific locus.

In agreement with the reviewer, we have modified our conclusions throughout the manuscript about the role of LmxESB1, as there are additional effects beyond the upregulation of amastins on chromosome 33. We now focus the discussion on the fact that LmxESB1 is important for the regulation of a subset of amastin genes on both chromosome 30 and 33 but not on chromosome 8.

We acknowledge that further experiments would be required to determine how LmxESB1 regulated amastin expression and to that end, we have attempted ChIP-seq with LmxESB1. However, we have not been able to get sufficient material for successful sequencing. We have included additional points within the discussion to indicate future experimental pathways; however, these are beyond the scope of this manuscript, which provides the first characterisation of LmxESB1.

* The authors focus on amastin and tuzin, which are of interest due to the role of ESB1 in T. brucei surface protein expression. However, other genes are altered - what are the 45 and 16 genes that show changed regulation, and how many of them are amastin and tuzin? This is not clear from the figures/text alone. The full results from the various transcriptomic analyses need to be provided.

We have now provided the full results of the various transcriptomic analyses (Tables S1-6). In Table S1, we list the genes up and downregulated after LmxESB1 deletion. Of the 16 genes downregulated there are few commonalities except for 5 genes associated with translation (LmxM.27.1710, LmxM.36.3880, LmxM.36.6980, LmxM.29.3240, LmxM.34.1430), which likely reflects the slow growth of these cells. We have included these additional details in the text.

Of the 45 upregulated genes, ~29% were either amastins (n=8) or tuzins (n=5) and were therefore of great interest to us. In addition, there were 3 cathepsin, 1 ascorbate peroxidase and 1 HASPa gene upregulated, all of which are also associated with amastigotes. Beyond the amastin, tuzin and cathepsin genes there were no obvious commonalities in terms of function of the upregulated genes. We have included these additional details in the text. As ever with an analysis of this nature in this organism, 18 of the 45 upregulated genes (40%) were annotated as hypothetical. Interestingly, 6 of these (LmxM.23.0420, LmxM.33.0020, LmxM.30.1165, LmxM.30.0460f, LmxM.09.0003, LmxM.27.2337) were upregulated by over 3-fold in a transcriptomic comparison of promastigotes versus amastigotes (Fiebig et al., 2015 - PMID: 26452044), further demonstrating that LmxESB1 is regulating a subset of transcripts upregulated in amastigotes.

Overall, we argue that this is strong evidence that the main function of LmxESB1is to ensure the regulation of amastin/tuzin expression, but we have acknowledged in the discussion that LmxESB1 perturbation also leads to changes in other amastigote-associated genes.

* The paper does not fully explore other possible roles for ESB1, to either rule them in or out. For example, the top gene with the highest fold change in the ESB1 deletion cell line is LmxM.34.0070 (deletion based on Fig 2D), a putative prohibitin, and this persistence with prolonged culture. ESB1 thus seems to have an important role in this genes repression but this is not explored?

The focus of Figure 2 was to identify candidates that were associated with the growth recovery of the LmxESB1 deletion mutant over time, this is distinct from the initial phenotype of ESB1 deletion relating to LmxESB1 function. Our rationale was that genes whose expression level had not changed after the initial deletion of LmxESB1 but then changed as the cell growth rate recovered were more likely to be involved in this compensation process than genes whose transcripts had changed initially. Hence, we focused on these genes, specifically those likely involved in mRNA regulation – RBP10, DRBD13, NIFP1.

© 2026. Published by The Company of Biologists under the terms of the Creative Commons Attribution License (https://creativecommons.org/licenses/by/4.0/).

In this analysis, we included genes whose expression had been up or downregulated during the time in which the growth of the ESB1 deletion mutant had recovered, with a significance threshold of p=0.05. This was a lower significance threshold than had been used for our initial analysis ($p=5.45 \times 10^{-6}$, which corrects for multiple comparisons) of ESB1 function, as this was a screen for potential candidates. Therefore, this analysis includes genes whose expression level changes were not considered significant in our initial functional analysis of ESB1 and LmxM.34.0070 (prohibitin) is one such example. It is possible the reviewer is thinking of LmxM.34.0030 not LmxM.34.0070, which in figure 2D is the most upregulated transcript. This encodes a pyruvate kinase but again does not reach our multiple comparison corrected threshold of $p=5.45 \times 10^{-6}$ at the initial week.

In terms of commonalities between the 21 genes that were upregulated during this time, 5 are associated with cell and mitochondrial protein production (LmxM.36.2020 (Chaperonin HSP60, mitochondrial precursor), LmxM.18.0740 (Elongation factor Tu, mitochondrial), LmxM.29.3240 (Glutamyl-tRNA synthetase), LmxM.13.1660 (Chaperonin TCP20), and LmxM.05.1200 (Prefoldin subunit)). The initial drop of these transcripts followed by an increase likely reflects the initial slower growth of the LmxESB1 deletion mutants, which then recovers. We have included this detail in the manuscript. For the downregulated transcripts, there are no obvious commonalities beyond the 12 amastin transcripts, with 18 (nearly half) of them annotated as hypothetical (Table S3). It is interesting to note that the transcript LmxM.09.0180 (ATG8), which was initially upregulated after LmxESB1 deletion has reduced in abundance. ATG8 is involved in autophagy and cell stress and its increase and then drop aligns with the mutant being stressed after initial deletion of LmxESB1 and then recovering after an extended period in culture.

We have now included these data in Table S3, so people can interrogate the genes themselves in more detail. In addition, as outlined in the responses above, we have provided additional detail about the transcripts whose expression changes immediately after LmxESB1 deletion that we believe to be more informative about LmxESB1 function than those presented in Figure 2D-E. This is complemented in the discussion in which we have broadened discussion to include the regulation of other amastigote-associated transcripts.

* Similarly, looking at the genome analysis figure there is a shared mutation on chromosome 9 for all the ESB1 knockout clones (Fig S2)? what is it?

This shared mutation is on Chr10 and is found in the gene LmxM.10.0470, which encodes GP63. The mutations is a SNP (missense-variant (c.238A>G)) Given it is within a gene family that encodes a surface protein, we did not think it likely had a role in regulating amastin, etc. expression.

* The claim that two proteins, RBP10 and NIFP1, are responsible for recovery of the ESB1 knockout lines, as they act in parallel with ESB1 to repress amastin and tuzins, could be convincing with additional experiments but currently isn't clear. RBP10 and NIFP1 can be tagged and localised, protein interactions established for example.

Based on this suggestion from the reviewer, we have now endogenously tagged RBP10, NIFP1 and DRBD13 on their C terminus with mNG (Figure S5). Both DRBD13 and NIFP1 were found in the cytoplasm, while there was less signal from the RBP10 cell line, with a reticulated signal indicative of low expression. The localisation of these proteins indicates that they do not directly interact with LmxESB1 but more likely have a post-transcriptional role in regulating amastin/tuzin and other transcripts abundance in the cytoplasm. We have included these points in the updated discussion.

Moreover, our identification of proteins, which do not directly interact with LmxESB1 but operate within the same pathway, shows the power of careful interrogation of transcript changes during deletion mutant recovery. This will open additional experimental avenues to unpick complex gene regulatory pathways, complementing approaches that focus on direct protein interactions.

* Similarly on Page 6, line 43: "Analysis of the deletion mutant transcriptomes showed that the slow growth of LmxRBP10 and LmxNIFP1 deletion mutants both correlated with an increase of amastin and tuzin transcripts" - no such growth data are provided and are there any genes with altered expression in all mutants? (other than the amastins and tuzin).

© 2026. Published by The Company of Biologists under the terms of the Creative Commons Attribution License (https://creativecommons.org/licenses/by/4.0/).

We have now provided the growth curves for these mutants (Figure 3A) and the associated transcriptomic data (Tables S4-6). In terms of up or downregulated genes there were none shared between all three mutants. If we restrict the comparison to RBP10 and NIFP1, we find one common downregulated gene LmxM.34.0520, which is a hypothetical protein and 30 upregulated genes. Of these 30 genes, 14 encoded amastins or tuzins and for the other 16 genes there was no obvious commonalities apart from the 3 genes encoding cathepsins and so we decided to focus the manuscript on the amastin and tuzin genes.

Minor comments

1. Fig 2D and E: The legend states the amastin genes are highlighted in blue but do not appear to be; equally, the RNA regulators chosen for investigation are not underlined.

We have changed the panel in this figure, carefully checking the x axis label, and it now matches the figure legend.

2. In Fig 3, the numbers of amastin and tuzin genes altered do not appear to match between A and C/D - was a different significance threshold used? Why?

We thank the reviewer for highlighting this issue - these plots were designed to show the statistically significant and >2x upregulated quadrant of the plots, however this did inadvertently lead to cropping of some key data points. Due to the very large p value range (even with the -log transform), we have provided the plots with the full y-axis range sufficient to cover all the points in Figure S4E. In addition, this aligns with the data in the new supplementary tables (Tables S4-6). In Figure 3, we have retained the plots, with the truncated scale, as they provide greater clarity of the changes near the significance threshold.

3. What % of the amastin genes in these regions (on chromosomes 33, 3, and 8) are altered? How would RBP10 act on specific chromosome regions? What does RBP10 localisation look like? Very few shared.

We thank the reviewer for this comment and have calculated the percentage of amastin genes on chr8, chr30 and chr33 that were altered after deletion of ESB1, RBP10 and NFIP1.

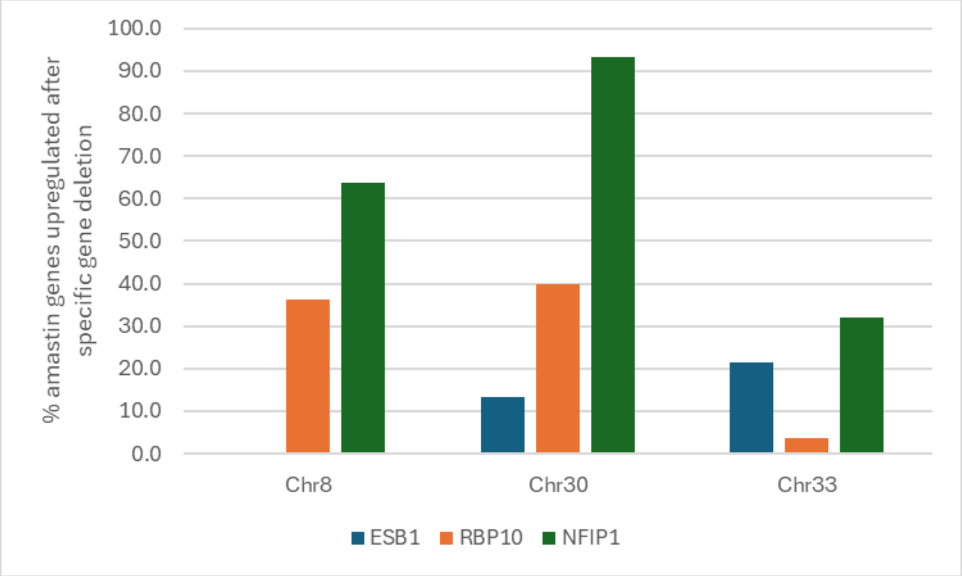

This shows that ESB1 affect a similar proportion of amastin genes on chr30 and chr33; however, it is quite clear that overall, the pattern of amastin changes between these 3 proteins is different. In light of this, we have now modified the text to say that ESB1 affects a subset of amastin genes and have expanded the focus to chr33 and chr 30. We have chosen not to include these percentages within the manuscript, as the genome around these amastin arrays is unlikely to be highly accurate, as the tandemly arrayed amastin genes are challenging to assemble. So, while we can draw

© 2026. Published by The Company of Biologists under the terms of the Creative Commons Attribution License (https://creativecommons.org/licenses/by/4.0/).

confident conclusions about the genomic loci of upregulated amastins, saying what proportion of amastins at that locus are upregulated is more ambiguous.

We have now endogenously tagged RBP10 (Figure S5A) and the protein localisation is faint but likely in the cytoplasm, as would be expected for an RNA binding protein and seen for the ortholog in T. brucei. Therefore, we do not envisage RBP10 acting on a specific chromosome region but likely on a specific cohort of mRNA transcripts. We have analysed the amastin UTRs but have not found any common motifs; however, this is again hampered by issues with sequence quality and annotation in these regions. We do not think it is likely that these would be simple motifs with a conserved sequence and are more likely to be based on sequence composition and 3D structure.

4. Page 7, lines 45-48 - typos in this sentence? The meaning is unclear.

We have reworked this sentence to clarify its meaning.

5. Typo on page 13, line 18 - pPLOT.

In this work we did use the pLPOT not the pPLOT tagging plasmid series and we have now clarified the methods to avoid confusion.

Second decision letter

MS ID#: jcs.264459R1

MS TITLE: Regulation of Leishmania surface coat proteins by the nuclear protein ESB1

AUTHORS: Jorge Adrián Arias del Angel; Ulrich Dobramysl; Laura Davidson; Richard J Wheeler; Jack D Sunter

ARTICLE TYPE: Research Article

Dear Dr Sunter,

We have now reached a decision on the above manuscript.

As you will see, the reviewers appreciated your response to their critiques and support publication. They have made only minor remaining suggestions that they believe will improve your final manuscript. I would like to give you the opportunity to address these remaining suggestions as you prepare a final version. I will then be pleased to accept your paper.

Reviewer 1

SUMMARY OF THE ADVANCE MADE IN THIS PAPER AND ITS POTENTIAL SIGNIFICANCE TO THE FIELD

SUGGESTIONS TO AUTHORS

Major comments [Please request additional experiments only if they are essential for supporting the conclusions; authors should be encouraged to highlight any claims that are preliminary or speculative, or to discuss any pitfalls or alternative interpretations in a 'Limitations' section]

Minor comments

After reading the authors' responses to my previous suggestions and the revised manuscript, I consider that the authors have addressed my suggestions properly and the manuscript has been improved.

© 2026. Published by The Company of Biologists under the terms of the Creative Commons Attribution License (https://creativecommons.org/licenses/by/4.0/).

There are two very minor points that authors may want to consider. These are:

1. Supplementary Table S1 (and also Table S3).
This Excel file contains valuable data, but there is a trouble: the ID codes are different to those used in the manuscript. This makes sense because the expression analyzes were done using a particular clone for which its genome and transcriptome were recently annotated; whereas in the manuscript, the ID codes used are the classical ones for L. mexicana genes. It would be recommendable to add a new column in which the classical ID are included (at least for the sheets with up- and down-regulated genes).

2. Lines 544-547. Check when Cf and Ci were measured.

Reviewer 2

SUGGESTIONS TO AUTHORS

This study identifies the interesting role of ESB1 as a repressor of surface protein encoding transcripts in Leishmania mexicana promastigote forms. This work is valuable for the field and highlights an apparently opposing role to the Trypanosoma brucei EBS1. I have only minor comments and feel the authors have made the previously requested changes to the figures and text. These revisions have improved the clarity of their results interpretation, included previously missing data, and increased the transparency of the findings. The results are thought provoking and will likely inform future investigations.

Minor comments

1. On line 123 the reference for the T. brucei work that localised TbEBS1 with polI/vex1/2 is missing.
2. Figure 1E is not referred to in the text.
3. In Tables S1-S6 there are tabs for "relevant" and "removed" genes. What are these and why are some removed? I'm assuming it is due to a quirk of the reference genome. These tables need legends and explanations of the tabs and columns in the excels to be clear for investigation by others.
4. In response to the comment about the genome changes, the authors explain why the shared mutation is on Chr10 in GP63 is unlikely to contribute to the change in phenotype after initial KO. I am in agreement, but think this could be briefly added to the manuscript text for clarity.
5. In figure 3E which shows the number of Tuzin genes in each KO, there only appear to be counts for chrom 33 or "others", but chrom 8 is also in the legend though not shown in the plot. The text mentions chr33 and chr8 only - is there an error somewhere here?

Second revision

Author response to reviewers' comments

Abstract limit

We have a strict limit for the summary or abstract of the paper to be under 180 words. Your abstract is currently 222 words. We would be grateful if you could reduce the length of your abstract before you re-upload your files.

We have reduced the length of the abstract.

Comments from the Reviewers:

Reviewer 1:

© 2026. Published by The Company of Biologists under the terms of the Creative Commons Attribution License (https://creativecommons.org/licenses/by/4.0/).

SUGGESTIONS TO AUTHORS

Minor comments

After reading the authors' responses to my previous suggestions and the revised manuscript, I consider that the authors have addressed my suggestions properly and the manuscript has been improved. There are two very minor points that authors may want to consider. These are:

1. Supplementary Table S1 (and also Table S3).

This Excel file contains valuable data, but there is a trouble: the ID codes are different to those used in the manuscript. This makes sense because the expression analyzes were done using a particular clone for which its genome and transcriptome were recently annotated; whereas in the manuscript, the ID codes used are the classical ones for L. mexicana genes. It would be recommendable to add a new column in which the classical ID are included (at least for the sheets with up- and down-regulated genes).

The gene IDs used in our analysis and reported in the supplementary tables are based on the genome sequence of the parental cell line which expresses Cas9 and T7 RNA polymerase. These gene IDs map directly to the classical ones so for clarity and future investigations we have converted them to the classical IDs i.e. LmxM379c.01.0010.1 becomes LmxM.01.0010.1. We have noted in the methods that there is a one-to-one correspondence for these gene IDs.

2. Lines 544-547. Check when Cf and Ci were measured.

Cf and Ci were measured every 24 hours. We ensured that Ci was ~$1x10^6$ cells/ml. We have clarified this in the methods.

Reviewer 2:

SUGGESTIONS TO AUTHORS

This study identifies the interesting role of ESB1 as a repressor of surface protein encoding transcripts in Leishmania mexicana promastigote forms. This work is valuable for the field and highlights an apparently opposing role to the Trypanosoma brucei EBS1. I have only minor comments and feel the authors have made the previously requested changes to the figures and text. These revisions have improved the clarity of their results interpretation, included previously missing data, and increased the transparency of the findings. The results are thought provoking and will likely inform future investigations.

Minor comments

1. On line 123 the reference for the T. brucei work that localised TbEBS1 with polI/vex1/2 is missing.

We have now included these references.

2. Figure 1E is not referred to in the text.

We have ensured Figure 1E is referred to in the text.

3. In Tables S1-S6 there are tabs for "relevant" and "removed" genes. What are these and why are some removed? I'm assuming it is due to a quirk of the reference genome. These tables need legends and explanations of the tabs and columns in the excels to be clear for investigation by others.

We have now provided legends for these tables and have renamed the tabs within the tables to clarify their contents. The removed genes referred to transcripts that aligned to genes not mapped

© 2026. Published by The Company of Biologists under the terms of the Creative Commons Attribution License (https://creativecommons.org/licenses/by/4.0/).

to the genome and/or had fewer than 20 reads in the parental sample. The relevant genes are those which are mapped and had 20 or more reads. We have renamed the tabs based on this explanation.

4. In response to the comment about the genome changes, the authors explain why the shared mutation is on Chr10 in GP63 is unlikely to contribute to the change in phenotype after initial KO. I am in agreement, but think this could be briefly added to the manuscript text for clarity.

We have now included this result in the text.

5. In figure 3E which shows the number of Tuzin genes in each KO, there only appear to be counts for chrom 33 or "others", but chrom 8 is also in the legend though not shown in the plot. The text mentions chr33 and chr8 only - is there an error somewhere here?

There were no transcripts from the tuzins encoded on chromosome 8 upregulated in any of the mutants we examined. Hence, there is no bar showing them on the plots in Figure 3E and the supplementary figures. We have now explicitly mentioned this in the text but have left the key on the plots to reinforce this.

Third decision letter

MS ID#: jcs.264459R2

MS Title: Regulation of Leishmania surface coat proteins by the nuclear protein ESB1

Authors: Jorge Adrián Arias del Angel; Ulrich Dobramysl; Laura Davidson; Richard J Wheeler; Jack D Sunter
Article Type: Research Article

Dear Dr Sunter,

I am happy to tell you that your manuscript has been accepted for publication in Journal of Cell Science, pending standard publication integrity checks.

© 2026. Published by The Company of Biologists under the terms of the Creative Commons Attribution License (https://creativecommons.org/licenses/by/4.0/).
